# circRNA-PTPN4 mediated regulation of FOXO3 and ZO-1 expression: implications for blood–brain barrier integrity and cognitive function in uremic encephalopathy

**DOI:** 10.1007/s10565-024-09865-6

**Published:** 2024-04-17

**Authors:** Yuhan Liu, Yanling Qin, Yanning Zhang

**Affiliations:** Department of Nephrology, General Hospital of the Northern Theatre, No. 83, Wenhua Road, Shenhe District, Shenyang, 110000 Liaoning Province People’s Republic of China

**Keywords:** UE, Blood–brain barrier, circRNA-PTPN4, miR-301a-3p, FOXO3, ZO-1, Tight junctions

## Abstract

Uremic encephalopathy (UE) poses a significant challenge in neurology, leading to the need to investigate the involvement of non-coding RNA (ncRNA) in its development. This study employed ncRNA-seq and RNA-seq approaches to identify fundamental ncRNAs, specifically circRNA and miRNA, in the pathogenesis of UE using a mouse model. In vitro and in vivo experiments were conducted to explore the circRNA-PTPN4/miR-301a-3p/FOXO3 axis and its effects on blood–brain barrier (BBB) function and cognitive abilities. The research revealed that circRNA-PTPN4 binds to and inhibits miR-301a-3p, leading to an increase in FOXO3 expression. This upregulation results in alterations in the transcriptional regulation of ZO-1, affecting the permeability of human brain microvascular endothelial cells (HBMECs). The axis also influences the growth, proliferation, and migration of HBMECs. Mice with UE exhibited cognitive deficits, which were reversed by overexpression of circRNA-PTPN4, whereas silencing FOXO3 exacerbated these deficits. Furthermore, the uremic mice showed neuronal loss, inflammation, and dysfunction in the BBB, with the expression of circRNA-PTPN4 demonstrating therapeutic effects. In conclusion, circRNA-PTPN4 plays a role in promoting FOXO3 expression by sequestering miR-301a-3p, ultimately leading to the upregulation of ZO-1 expression and restoration of BBB function in mice with UE. This process contributes to the restoration of cognitive abilities.

**Graphical Abstract:**

1. The circRNA-PTPN4/miR-301a-3p/FOXO3 axis is identified as a key regulator of blood–brain barrier integrity and cognitive function in uremic encephalopathy.

2. circRNA-PTPN4 sequestration of miR-301a-3p enhances FOXO3 expression, leading to upregulation of ZO-1 and improved endothelial permeability.

3. Overexpression of circRNA-PTPN4 in uremic mice restores cognitive abilities and reduces neuronal loss and inflammatory infiltration.
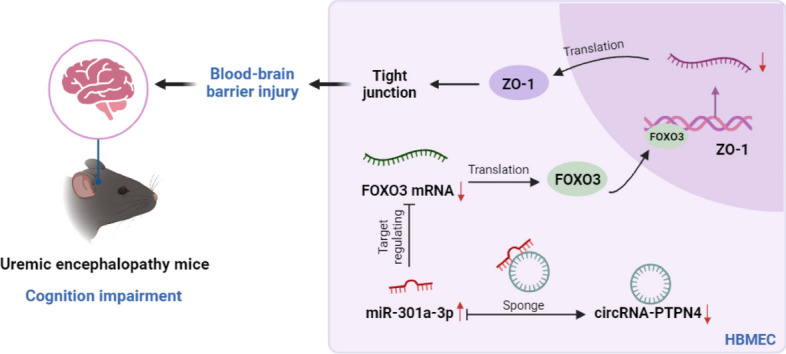

**Supplementary Information:**

The online version contains supplementary material available at 10.1007/s10565-024-09865-6.

## Introduction

Uremic encephalopathy (UE) is a severe complication that could arise when chronic kidney disease advances to the uremic stage, predominantly impacting the central nervous system of patients (Rosner et al. 2022; Yanai et al. 2019). Common symptoms of this condition include compromised consciousness, malfunctioning motor nerves, and behavioral and cognitive impairments (Gregory et al. 2017; Aono et al. 2021). Although UE substantially impacts patients’ quality of life and prognosis, its exact pathogenesis remains unclear. This lack of clarity presents a challenge in the search for effective treatment methods.

In recent years, there has been progress in the study of non-coding RNA (ncRNA) in biomedicine (Hill and Tran 2021; Yan and Bu 2021; Panni et al. 2020; Anastasiadou et al. 2018). Of note, ncRNAs, particularly microRNAs (miRNAs) and circular RNAs (circRNAs), are recognized to confer crucial functions in proliferative, migratory, and apoptotic capabilities of cancers (Yan and Bu 2021; Li et al. 2020a; Ali et al. 2021). The roles and mechanisms of ncRNAs in neurological diseases, including UE, have received attention from researchers (Visitchanakun et al. 2020).

The blood–brain barrier (BBB) is vital in shielding the brain from harmful substances and serves as the principal impediment for drugs to penetrate the brain (Zhao et al. 2022; Candelario-Jalil et al. 2022; Sweeney et al. 2019). Various neurological disorders have been found to cause impairment in the function of the BBB, with UE being particularly notable (Candelario-Jalil et al. 2022; Faucher et al. 2023; Hosoya and Tachikawa 2011). Hence, grasping the molecular dynamics influencing the BBB function is essential. Such knowledge is pivotal for delving into the pathogenesis of UE and devising appropriate treatment methodologies.

Based on the background and understanding provided, this study aims to elucidate ncRNA’s pivotal role and underlying mechanisms in UE using high-throughput sequencing technology. Specifically, our investigation was centered on exploring the involvement of the circRNA-PTPN4/miR-301a-3p/FOXO3 axis in this process, as well as its contribution to the onset and progression of UE by affecting the function of the BBB. Through these studies, we aim to provide novel insights and strategies for treating UE, resulting in tangible clinical benefits for patients.

## Materials and methods

### Ethics statement

The research involving animals was carried out in alignment with established ethical standards for animal experimentation. Approval for the animal study protocol was granted by the Institutional Animal Care and Use Committee at the General Hospital of the Northern Theatre.

### Sample preparation for sequencing

The isolation of total RNA from brain and kidney tissues of both normal mice (*n* = 3) and uremic mice (*n* = 3) was accomplished using Trizol reagent (Thermo). Quantification of RNA, along with assessments of its purity and structural integrity, was conducted through the utilization of a Qubit® 2.0 Fluorometer® in conjunction with a Qubit® RNA Assay Kit for concentration measurements, an IMPLEN Nanodrop spectrophotometer for evaluating RNA purity, and an RNA Nano 6000 Assay Kit employed on an Agilent Bioanalyzer 2100 system for determining the structural integrity of the RNA samples. The measurements yielded results indicating that RNA concentration was equal to or greater than 20 ng/μL, purity exceeded an OD260/280 ratio of 2.0, and integrity showed a RIN value equal to or greater than 7.0 with a 28S/18S ratio equal to or greater than 1.0 (Arunachalam et al. 2022).

### High-throughput transcriptome sample sequencing

For the preparation of RNA samples, 3 μg of total RNA from each sample served as the input for cDNA library preparation using the NEBNext® Ultra™ RNA Library Prep Kit for Illumina® (NEB, E7435L, Beijing, China). The library quality was assessed based on indexing and clustering using the TruSeq PE Cluster Kit v3 cBot HS (Illumina) on the cBot system. Sequencing was conducted on the Illumina HiSeq 550. Moreover, the quality control was performed using FastQC v0.11.8, with data preprocessing by Cutadapt v1.18 to remove adapter and poly(A) sequences. Reads with more than 5% N bases were discarded via a Perl script, and those with base quality over 20 were selected with FASTX Toolkit v0.0.13. BBMap software was used for read pairing, and HISAT2 v0.7.12 aligned the high-quality reads to the mouse genome, ensuring data integrity and preparation for analysis (Deng et al. 2020; Peng et al. 2019).

### miRNA high-throughput sequencing and quality control

For the small RNA library construction, 3 μg of total RNA from each sample served as the starting material for small RNA library construction using the NEBNext® Multiplex Small RNA Library Prep Set for Illumina® (E7300S, NEB), with unique index codes assigned for sample identification. The method involved direct ligation of NEB 3’ SR Adaptors to the 3’ ends of miRNAs, siRNAs, and piRNAs, conversion of single-stranded adaptors to double-stranded DNA, and first-strand cDNA synthesis using M-MuLV Reverse Transcriptase. PCR amplification used LongAmp Taq 2X Master Mix with the SR Primer serving as the Illumina and index primer. PCR products were purified on an 8% polyacrylamide gel, isolating DNA fragments of 140–160 base pairs, and resuspended in 8 μL elution buffer. The library’s quality was assessed with the Agilent Bioanalyzer 2100, followed by clustering with the TruSeq SR Cluster Kit v3-cBot-HS (GD-401–3001, Illumina) and sequencing on the Illumina HiSeq 2500/2000 platform to produce 50 bp reads.

The raw data presented in Fastq format underwent processing through the deployment of Perl and Python scripts. This crucial step entailed the exclusion of reads characterized by the presence of poly-N sequences, contamination with a 5’ adaptor, absence of a 3’ adaptor or insert tag, presence of homopolymeric runs of A, T, G, or C, and those of inferior quality, thereby resulting in the acquisition of purified data. Furthermore, critical metrics, including Q20 and Q30 scores, and the GC content pertaining to the raw data were meticulously computed. Following the initial purification, reads falling within a predetermined length range were isolated from the cleansed reads for further analytical procedures. The construction of a reference genome index was achieved via Bowtie2 version 2.2.8, allowing for the alignment of paired-end purified reads against the reference genome. The identification of established miRNAs was facilitated through the use of corresponding miRNA tags. Additionally, the potential miRNAs were discerned by referencing miRBase 20.0, utilizing the capabilities of mirdeep2 and srna-tools-cli software for this purpose (Ma et al. 2020).

### CircRNA high-throughput sample sequencing

For circRNA sequencing, 5 μg of total RNA was prepared, with ribosomal RNA removal via the Epicentre Ribo-zero™ kit and ethanol precipitation to clear residuals. The RNA was treated with RNase R to degrade linear RNAs before constructing sequencing libraries using the NEBNext® Ultra™ Directional RNA Library Prep Kit for Illumina®, following the manufacturer’s instructions. This procedure included RNA fragmentation, first-strand cDNA synthesis using random hexamers and M-MuLV Reverse Transcriptase, followed by second-strand synthesis with DNA Polymerase I and RNase H, substituting dUTP for dTTP. After DNA end adenylation and adaptor ligation, cDNA fragments (150–200 bp) were purified with the AMPure XP system and PCR-amplified, with the final products assessed on the Agilent Bioanalyzer 2100. The indexed samples were then clustered and sequenced on the Illumina HiSeq 4000, generating 150 bp paired-end reads.

Preprocessing of raw Fastq data involved removing adaptor sequences, poly-N, and low-quality reads via Perl scripts, leading to purified data. Quality metrics (Q20, Q30, GC content) of this data were calculated. The cleaned data underpinned further analysis, with reference genome and gene annotation files sourced directly from genome databases. A reference genome index was created with Bowtie2 v2.2.8 for read alignment. Circular RNAs were identified using find_circ and CIRI2 software, and raw counts were normalized to TPM, with normalized expression calculated as (readCount*1,000,000)/libsize, where libsize is the sum of circRNA read counts (Ma et al. 2019).

### Sequencing data analysis

The R software package "Limma" was employed for the analysis of miRNAs and circRNAs that exhibited differential expression between control and uremic groups, adopting thresholds of absolute log fold change (|logFc|) greater than 1 and a *P*-value less than 0.05. For the selection of differentially expressed genes (DEGs), the criteria set were an absolute log fold change (|logFC|) exceeding 1 and a *P*-value below 0.001. Visualization of DEGs was achieved through the "heatmap" package for heatmaps and "ggplot2" for constructing volcano plots to illustrate the variations in gene expression. The construction of Venn diagrams to display gene overlap utilized the "vennDiagram" package. Functional enrichment analyses, including GO and KEGG pathways, were conducted using the "clusterProfiler" package. Protein–protein interaction (PPI) networks were explored using the STRING database online, while circRNA-miRNA interaction predictions were made via the ERCOI web resource. Predictions of miRNA target genes were facilitated through both the miRmap and Tarbase databases (Qi et al. 2022).

### In *vitro *cell culture

Human brain microvascular endothelial cells (HBMECs) (CRL-3245) were maintained in a culture medium consisting of DMEM/F12, enriched with 40 μg/ml endothelial cell growth supplement (ECGS) and 10% FBS. Meanwhile, HEK-293 T cells (CRL-3216), utilized in this research, were sourced from ATCC, United States. These cells were maintained at optimal growth conditions in a Thermo Fisher incubator set at 37 °C with an atmosphere of 5% CO_2_. The culture media components, FBS and DMEM/F-12, were acquired from Gibco (Chen et al. 2012).

### Cell transfection

Lentiviral particles were packaged in HEK-293 T cells via transfection with the target plasmid along with the auxiliary plasmids pMD2.G (12,259, Addgene) and psPAX2 (12,260, Addgene) using the Lentiviral Packaging Kit (V48820, Invitrogen). Then, 48 h post-transfection, supernatants were collected, concentrated using Lentivirus Concentration Solution (Takara), and stored at -80 °C. Cells in the logarithmic growth phase were digested with trypsin and seeded at a density of 1 × 10^5^ cells per well in 6-well plates. Then, 48 h following infection, 10 μg/mL puromycin (540,222, Sigma-Aldrich) was used for selection, maintained for at least 1 week to establish stably transfected cell lines. The construction of target plasmids was undertaken by Shanghai Hanheng Biotechnology Co., Ltd., with sh-RNA and si-RNA sequences detailed in Table S1.

Transfection of mimics-NC/miR-301a-3p-mimics and inhibitors-NC/miR-301a-3p-inhibitors was performed using lipofection, with both mimics and inhibitors procured from Sangon Biotech (Shanghai, China). The sequences were as follows: mimics-NC: UUGUACUACACAAAAGUACUG; miR-301a-3p-mimics: CAGUGCAAUAGUAUUGUCAAAGC; miR-301a-3p-inhibitors: GCUUUGACAAUACUAUUGCAC. For lipofection-mediated transfection, 5 × 10^5^ cells were seeded in 6-well plates, and at 70–90% confluency, transfection was carried out using Lipofectamine™ 3000 (Thermo Fisher, L3000150). Then, 2 to 4 days post-transfection, the cells were used for identification and further analyses (Zeng et al. 2023; Nishiyama et al. 2022; Ouyang et al. 2020).

Cell groups for the experiment were organized as follows: for circRNA-PTPN4: si-NC, si-circ-1, si-circ-2, oe-NC, oe-circ; for miR-301a-3p: Mock, inhibitor NC, miR-301a-3p inhibitor, mimics NC, miR-301a-3p mimics; for FOXO3: sh-NC, sh-FOXO3-1, sh-FOXO3-2, oe-NC, oe-FOXO3, oe-circ + sh-FOXO3, si-circ + oe-FOXO3.

### Gene and protein expression profiling

Total RNA was isolated from both tissues and cellular samples utilizing the Trizol reagent provided by Thermo Fisher Scientific, and subsequently reverse-transcribed into cDNA with the First Strand cDNA Synthesis Kit (D7168L, Beyotime, Shanghai, China). In the case of miRNAs, cDNA synthesis was facilitated through a PolyA Tailing Kit (Sangon Biotech, Shanghai, China), enabling the generation of miRNAs appended with PolyA tails. RT-qPCR was performed using an RT-qPCR Kit (Q511-02, Vazyme Biotech, Nanjing, China) following the manufacturer’s instructions. Primer sequences were designed and supplied by Sangon Biotech (Shanghai, China), with details in (Table S2). GAPDH served as the internal reference for mRNA, and U6 for miRNA. Gene expression quantification was achieved using the 2-ΔΔCt method (Ayuk et al. 2016).

For protein extraction from tissues and cells, RIPA lysis buffer containing 1% PMSF (Beyotime) was used. SDS-PAGE gels of 8%-12% were prepared according to the size of the target protein bands, and proteins were separated by electrophoresis. Proteins immobilized on the gel were subsequently transferred onto a PVDF membrane. The membrane was incubated with a 5% solution of non-fat milk at ambient temperature for a duration of 1 h. Primary antibodies (Table S3) were added and incubated overnight at 4 °C. HRP-conjugated goat anti-rabbit IgG secondary antibody (ab6721, 1:2000, Abcam, UK, and Cell Signaling Technology) was applied and incubated for 1 h at room temperature. The bands were visualized using ECL solution (1,705,062, Bio-Rad) on an Image Quant LAS 4000C gel documentation system (GE). As normalized to GAPDH, band intensities were quantified using ImageJ software to determine protein levels (Ban et al. 2019).

### Assessment of cell viability, proliferation, migration, and apoptosis

The evaluation of cell viability was conducted utilizing the Cell Counting Kit-8 (CCK-8, Beyotime). Cells, resuspended and adjusted to a density of 1 × 10^3^ per well, were seeded into 96-well plates and cultured overnight. Subsequent to culture intervals of 24, 48, and 72 h, each well received 10 μL of the CCK-8 solution, which was then incubated for 1 h prior to the assessment of optical density at 450 nm using a microplate spectrophotometer (E8051, Promega) (Yang et al. 2016).

For proliferation rates, the EdU labeling assay was utilized. Following seeding in 24-well plates, cells were treated with 10 µmol/L EdU (Beyotime) and allowed to incubate for 2 h. Subsequently, the cells were immobilized using 4% paraformaldehyde, rendered permeable with 0.5% Triton X-100, and subjected to staining via the EdU click reaction protocol. DAPI staining was applied to visualize nuclei. Fluorescence microscopy (FV-1000/ES, Olympus, Japan) quantified the percentage of EdU-positive cells in random fields (Yang et al. 2022).

Wound healing assays were conducted by seeding cells in 6-well plates until reaching 90–100% confluence, followed by creating a scratch with a pipette tip. Images were taken at 24 h to measure migration distances using Image J software, calculating relative migration rates (Chen et al. 2022).

TUNEL assays was used to detect apoptosis with the Beyotime TUNEL Apoptosis Detection Kit. Fixed and permeabilized cells were incubated with a TUNEL reaction mixture and counterstained with DAPI. Fluorescent microscopy identified TUNEL-positive cells, and the apoptosis rate was calculated by counting positive cells in five random fields per sample (Cheyuo et al. 2012). Each experiment was replicated three times to ensure the reliability and reproducibility of the findings.

### Luciferase activity assay

The cDNA fragments of circRNA-PTPN4 and FOXO3 containing miR-301a-3p binding sites, along with a DNA fragment of ZO-1 harboring a FOXO3 binding site, were cloned into the pmirGLO vector. Mutated versions of these fragments, synthesized through site-directed mutagenesis, were also inserted into the pmirGLO vector. The sequences for each construct were as follows: circRNA-PTPN4 Wild Type (Wt): AAAACUUCAGCACUGUUGCACUU; circRNA-PTPN4 Mutant (Mut): AAAACUUGUGCUGAGAACGUGAU; FOXO3 Wt: GCCGAGAUCAUGCCAGUGCACUC; FOXO3 Mut: GCCGAGAUCAUGCCAGUGCACUC; ZO-1 Wt: TGTAAACA; ZO-1 Mut: ACATTTGT. Based on lipofection, HEK293T cells were co-transfected with either circRNA-PTPN4-Wt/Mut or FOXO3-Wt/Mut recombinant vectors and either mimics NC or mimics miR-301a-3p; ZO-1 Wt/Mut recombinant vectors with either oe-NC or oe-FOXO3. After a 48-h incubation, the Dual-Luciferase® Reporter Assay System (E1910, Promega) facilitated the measurement of reporter gene activity, using Renilla luciferase as an internal reference (Jin et al. 2020).

### RNA/DNA pull-down assay

Biotinylated constructs of circRNA-PTPN4 WT/Mut, FOXO3 WT/Mut, and ZO-1 WT/Mut (KeyGEN BioTECH, Wuhan, China) were transfected into HBMEC cells for 48 h. Cells were harvested, washed with PBS, and lysed. Lysates were incubated with RNase-free BSA and yeast tRNA-precoated streptavidin magnetic beads (Merck, LSKMAGT) at 4 °C overnight. Following washes with lysis, low-salt, and high-salt buffers, bound RNAs were purified using Trizol and analyzed for miR-301a-3p and FOXO3 enrichment via RT-qPCR (Luan et al. 2018).

### ChIP-qPCR

Cells fixed with 1% formaldehyde were sonicated, generating appropriately sized DNA fragments, and centrifuged. Supernatants were incubated with anti-rabbit IgG (negative control) or anti-FOXO3 antibody (Abcam, ab70315, UK) overnight at 4 °C. DNA–protein complexes were precipitated, decrosslinked at 65 °C overnight, and purified. ChIP-qPCR products were analyzed on a 3% agarose gel, with primer sequences in Table S4 (Yang et al. 2016).

### Fluorescence in situ hybridization (FISH)

Probes for circRNA-PTPN4 and miR-301a-3p were obtained from Sangon Biotech (Shanghai, China). After denaturing and fixation, cells were treated with sodium bisulfite and proteinase K, followed by dehydration through an ethanol series. Post-hybridization overnight at 37 °C in a humidified chamber, slides were washed and counterstained with DAPI. Fluorescence microscopy observed the hybridization signals (Ye et al. 2018).

### Transendothelial electrical resistance (TEER) measurement

TEER was assessed prior to FITC-dextran permeability evaluation, following previously described protocols. Briefly, the culture medium in both dishes and Transwells was replaced with 0.1 M KCl. The EndOhm chamber’s cap was inserted at the top of the chamber, connecting the Transwell with a connector cable, and resistance was measured using an EVOM resistance meter (World Precision Instruments, Sarasota, FL). A new Transwell containing 0.1 M KCl, devoid of cells, served as a blank control (Zhao et al. 2019).

### FITC-dextran endothelial permeability assay

For FITC-dextran permeability assessment, HBMECs in the logarithmic growth phase were seeded at a density of 1 × 10^5^ cells onto the upper chamber of 24-well Transwell plates (3524, Corning). Each chamber was supplemented with 100 µL and 600 µL of medium, respectively, and incubated at 37 °C in a 5% CO_2_ incubator. Upon reaching confluence, 1 mg/mL FITC-dextran (Sc-263323, SANTA CRUZ) was introduced and incubated at 37 °C for 5 min in a 5% CO_2_ incubator. A 200 µL sample of the baseline medium was taken to determine the baseline value. After an additional 24-h incubation with the supplemented medium, another 200 µL of the baseline medium was collected to measure FITC fluorescence intensity using a microplate reader (Wang et al. 2021).

### UE mouse model

A total of 126 male C57BL/6 mice, aged 5–6 weeks (sourced from Vital River Laboratory Animal Technology Co. Ltd., Beijing, China), were maintained under pathogen-free conditions at 26–28 °C and 50–65% humidity. An adenine-rich diet was utilized to establish a mouse model of UE. Mice were initially fed a diet containing 20% casein (C7906-5G, Sigma) for 7 days, followed by a diet supplemented with 0.2% adenine (Adenine code A0230000, Sigma) mixed with casein for another 7 days to induce tubular injury. This process was continued for 7 weeks with a diet containing 0.15% adenine. Control mice were fed a standard diet. After 9 weeks, blood samples from the tail vein were tested using a creatinine assay kit (DICT-500, BioAssay Systems) and a urea nitrogen assay kit (BC1535, Solarbio). Elevated creatinine and urea nitrogen levels in the uremic mice compared to the normal mice indicated successful model establishment.

From week 8 onwards, mice underwent daily Y-maze and Morris water maze tests to assess their cognitive functions. Upon exhibiting cognitive impairments, the mice were deeply anesthetized with isoflurane and perfused with ice-cold PBS. Brain and kidney tissues were collected for sequencing and histological analysis. Stereotaxic microinjections (Dorsal -0.26 cm, Lateral -0.15 cm, Anterior -0.02 cm) delivered 4 μL of lentivirus at a titer of 2 × 10^8^ units/mL into the mouse ventricles at a rate of 1 μL/min for 7 consecutive days.

Animal grouping for sequencing involved random assignment into a normal group (Normal) and a UE group (Uremia), with 3 mice in each group. For in vivo experiments, mice were divided into the normal group (Normal), UE group (Uremia), UE plus circRNA-PTPN4 overexpression and silence control group (oe-circ + sh-NC), and UE plus circRNA-PTPN4 overexpression and FOXO3 silencing group (oe-circ + sh-FOXO3), with 30 mice in each group. Fifteen mice from each group underwent Evans blue staining (Kim et al. 2021; Fang et al. 2023; Zhang and Liu 2023).

### Y-maze test

The apparatus consisted of a maze with a central starting zone and three branching arms, each measuring 45 cm in length, 10 cm in width, and 15 cm in height, with reward food placed at the end of each arm. Mice were initially allowed to adapt to the experimental setup freely. They were then placed in the starting zone, with their initial positions standardized, and their behavior, including exploration, dwell time, and arm entry frequency, was recorded using a camera. After familiarization with the maze layout and food locations, the testing phase evaluated spatial learning and memory by altering the food’s placement to observe if mice could locate the correct position. Data analysis included exploration time, the number of entries into different arms, and the ability to find the reward location (Sun et al. 2021).

### Morris water maze test

The experiment was conducted in a 120 cm diameter, 50 cm high pool filled with clear, body-temperature water. The test included a 15 cm diameter circular escape platform submerged 2 cm below the water’s surface and camouflaged with opaque color. Mice were acclimatized to reduce stress before the trial, involving familiarization with the lab and pool. Experiments commenced from varying starting points around the pool, with mouse behavior meticulously recorded. Training sessions enabled mice to locate the submerged platform, initially marked with cues later removed to assess if mice could independently find the platform. Metrics such as escape latency, occupancy in the target quadrant, platform crossings, and total swimming distance were statistically evaluated (Wei et al. 2021).

### Histological and immunological analysis

For hematoxylin and eosin (H&E) staining, coronal sections of brain tissues, 20 µm thick, were stained using H&E, with incubation of 2 min for hematoxylin and 1 min for eosin. Sections underwent dehydration and permeabilization before microscopic observation (BX63, Olympus, Japan) (Li et al. 2020b).

For immunofluorescence assay, fixed cells or tissue sections were treated to allow penetration of primary antibodies targeting ZO-1 (ab307799, Abcam, UK), Occludin (ab216327, Abcam, UK), Claudin-5 (ab131259, Abcam, UK), and CD31 (Sc-376764, SANTA CRUZ, US), followed by incubation with Alexa Fluor-conjugated secondary antibodies and DAPI staining. Confocal microscopy facilitated the visualization of these markers (Li et al. 2019).

The immunohistochemistry protocol entailed the use of antibodies specific to NeuN (ab177487, Abcam), TNF-α (ab307164, Abcam), and IL-1β (ab283818, Abcam). Following secondary antibody application and SABC amplification, the DAB chromogen revealed the localization of target proteins, which was counterstained with hematoxylin. Observations were made under an upright microscope (BX63, Olympus, Japan) (Li et al. 2020b).

### Evans blue assay

To assess vascular leakage, mice were intravenously injected with 100 µL of 2% Evans Blue (E2129, Sigma Aldrich, MO). Then, 2 h post-injection, mice were euthanized and perfused with PBS. Brain tissues were then dissected and examined under a stereo microscope for horizontal and coronal sections. After removing the cerebellum and olfactory bulbs, half-brains were weighed, homogenized in 1 mL formamide (F274368, Aladdin, Shanghai, China), and incubated at 60 °C overnight. Brain homogenates were centrifuged at 14,000 rpm for 30 min, and the supernatant containing Evans Blue was transferred to a 96-well plate, with 200 µL per well, including replicates. Optical density at 620 nm was measured using a pre-warmed microplate reader (Li et al. 2022).

## Statistical analysis

Bioinformatics results were analyzed using R version 4.2.1, while other analyses were conducted using SPSS version 26.0 (IBM). Quantitative data were presented as mean ± standard deviation. Normality and homogeneity of variance tests were initially performed. Groups conforming to normal distribution and homogeneity of variance were analyzed using unpaired or paired t-tests for two-group comparisons, while one-way ANOVA or repeated measures ANOVA were used for multi-group comparisons. A *P*-value < 0.05 was considered statistically significant.

## Results

### Identification of miR-301a-3p as a key miRNA associated with BBB injury in UE

**Fig. 1:**
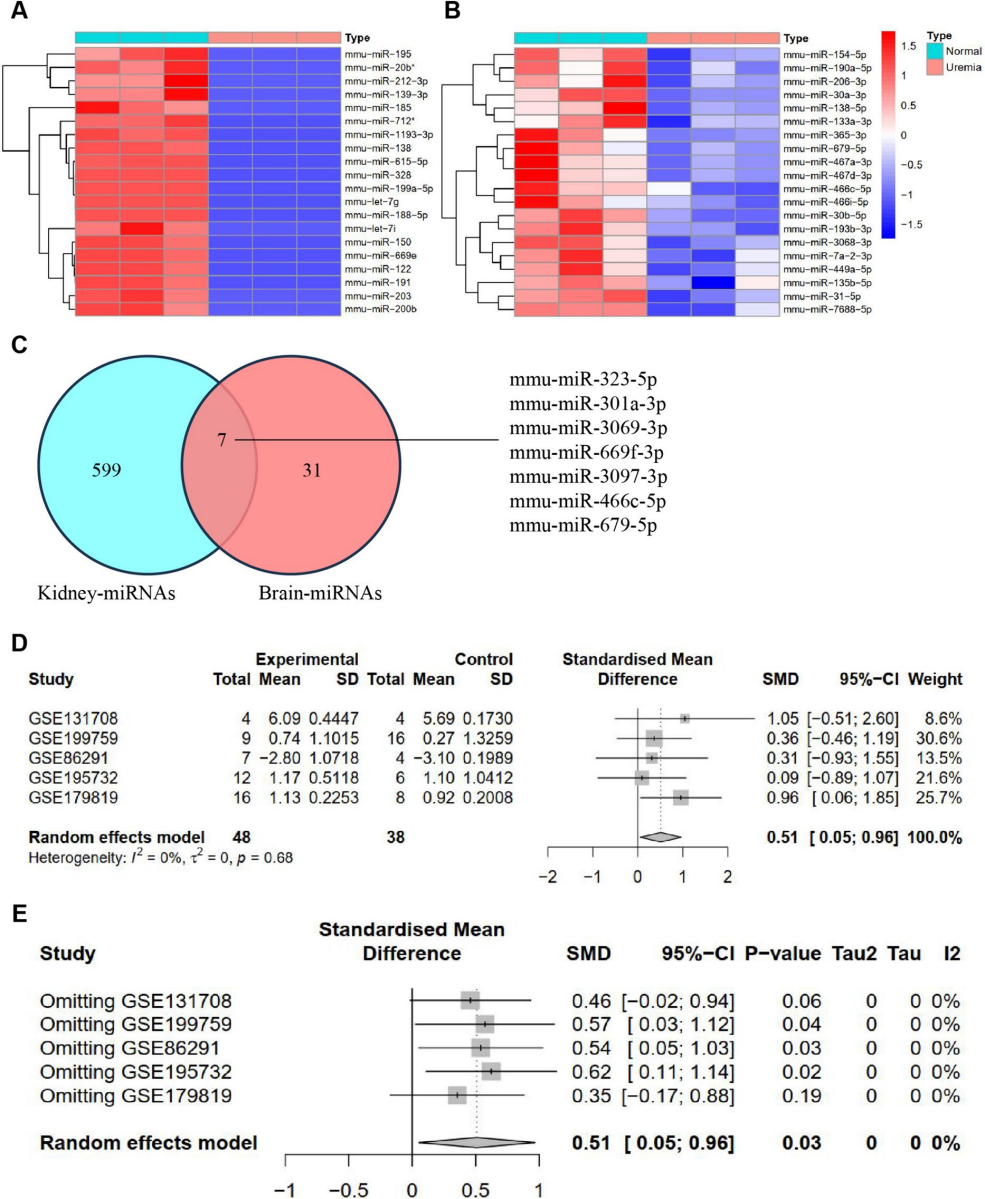
Differential expression analysis of miRNAs in UE mice using ncRNA-seq. Note: (**A**) Heatmap of DEMs in mouse kidney tissues from Normal (*n* = 3) and Uremia (*n* = 3) groups, with red representing upregulated genes and blue representing downregulated genes; (**B**) Heatmap of DEMs in mouse brain tissues from Normal (*n* = 3) and Uremia (*n* = 3) groups, with red representing upregulated genes and blue representing downregulated genes; (**C**) Venn diagram analysis of DEMs between kidney and brain tissues; (**D**) Forest plot of miR-301a-3p expression differences between Normal group and BBB injury group, with the middle vertical line indicating an ineffective line (SMD = 0), suggesting no statistical association between the study factors and outcome. Each horizontal line represents the 90% CI of the study, while the small square at the center of the line represents the point estimate of MD, with the square’s size reflecting the study’s weight. If the 95% CI of a certain study does not cross the ineffective line (i.e., 95% CI does not cross 0), it could be considered that there is a statistical association between the study factors and outcome; (**E**) Sensitivity analysis results of the stepwise exclusion method for miR-301a-3p; (*n* = 3 for each group). SMD: standard mean difference; 90% CI: 95% confidence interval

To examine the molecular mechanisms of miRNAs in BBB injury in UE, we analyzed miRNA expression levels in kidney and brain tissues from normal mice (*n* = 3) and mice with UE (*n* = 3) using high-throughput sequencing of ncRNAs (ncRNA-seq). We identified 617 differentially expressed miRNAs (DEMs) in kidney tissue, which we call kidney miRNAs. Among them, 132 miRNAs were downregulated, and 485 miRNAs were upregulated, based on the criteria of |logFc|> 1 and *P* < 0.05 (Fig. 1A). A total of 38 DEMs, referred to as brain-miRNAs, were identified in brain tissue using the criteria of |logFc|> 0.5 and *P* < 0.05. Among these miRNAs, 26 were downregulated, while 12 were upregulated (Fig. 1B). Subsequently, we performed an intersection analysis on the DEMs from both groups, revealing 7 miRNAs with differential expression common to the kidney and brain tissues of mice with UE (Fig. 1C). The expression trends of mmu-miR-323-5p, mmu-miR-301a-3p, and mmu-miR-669f-3p were found to be upregulated in mouse kidney and brain tissues, while mmu-miR-669f-3p showed a downregulation.

To identify the key miRNAs involved in BBB injury in UE, we retrieved BBB injury-related datasets (GSE86291, GSE131708, GSE179819, GSE195732, GSE199759) from the GEO database. Detailed information about these datasets is provided in Table S5. We validated the expression of mmu-miR-323-5p, mmu-miR-301a-3p, and mmu-miR-669f-3p through meta-analysis. The results revealed that miR-301a-3p was the only miRNA expressed in all five datasets. Compared to the Normal group, miR-301a-3p showed upregulation in the BBB injury group, indicating its potential role in the molecular mechanisms of BBB injury (Fig. 1D).

**Fig. 2:**
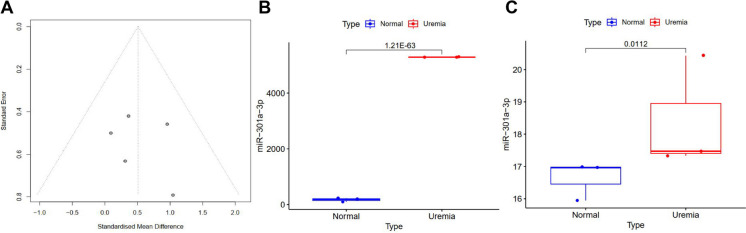
High expression of miR-301a-3p in mice with UE. Note: (**A**) Funnel plot of publication bias in meta-analysis data of miR-301a-3p, with a smaller bias of the "funnel" relative to the central dotted line indicating a more uniform data distribution; (**B**) Expression levels of miR-301a-3p in mouse kidney tissues from Normal group (*n* = 3) and Uremia group (*n* = 3) as determined by ncRNA-seq; (**C**) Expression levels of miR-301a-3p in mouse brain tissues from Normal group (*n* = 3) and Uremia group (*n* = 3) as determined by ncRNA-seq; (*n* = 3 for each group)

Moreover, sensitivity and subgroup analyses were conducted using the one-by-one elimination method on the research results included in this study. The findings indicate that the MD value did not change, suggesting the reliability of the meta-analysis results (Fig. 1E; Figure S1). The results of bias detection revealed that the data from the meta-analysis were evenly distributed within the funnel plot, suggesting the absence of any publication bias (Fig. 2A).

Finally, we conducted calculations to analyze the expression level of miR-301a-3p in the sequencing data. The results demonstrated upregulation of miR-301a-3p expression levels in the kidneys and brain tissues of mice with UE (Fig. 2B-C). These results suggest a high expression of miR-301a-3p in mice with UE.

### Effect of the circRNA-PTPN4/miR-301a-3p/FOXO3 axis on BBB damage during UE

**Fig. 3:**
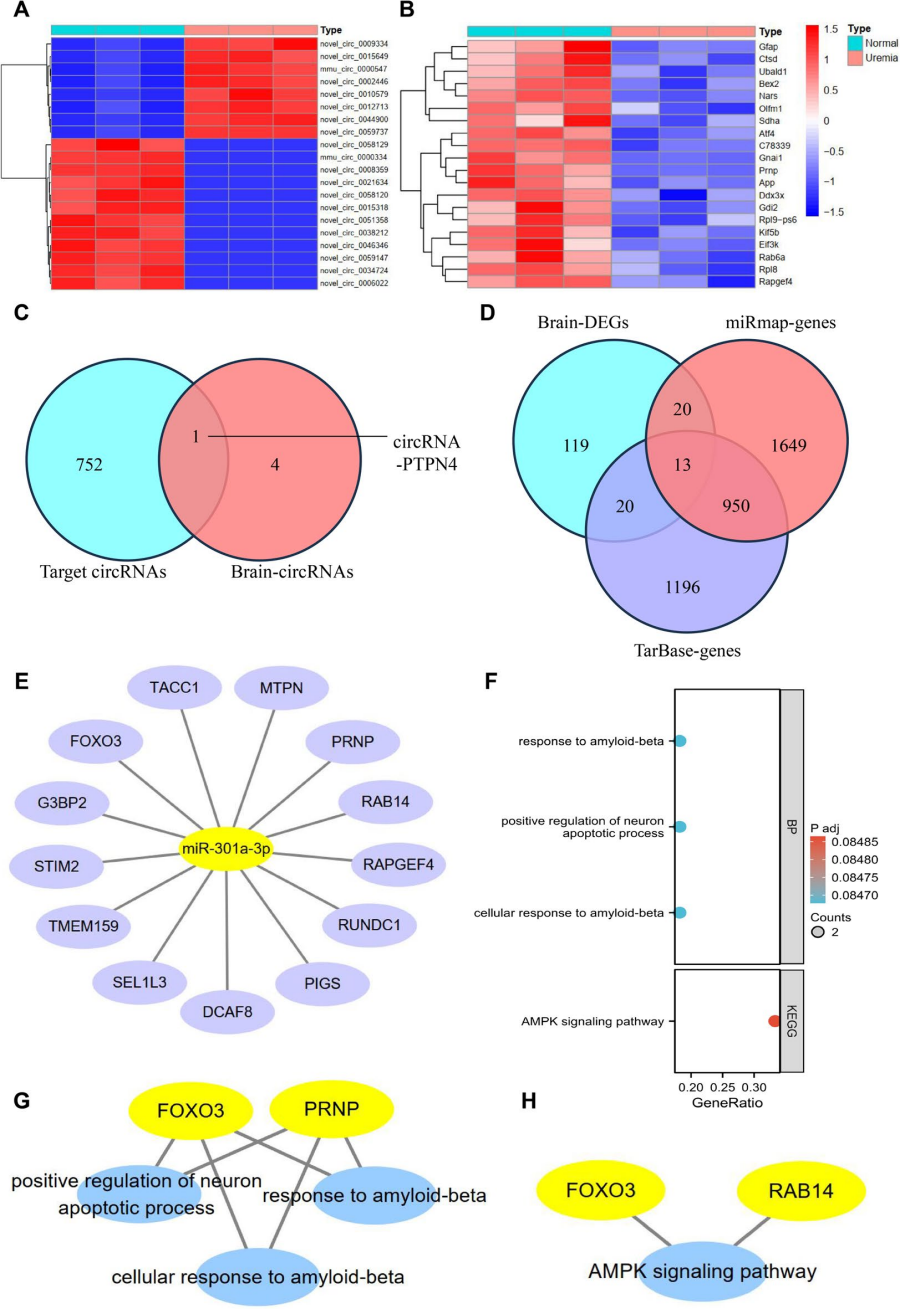
Identification of circRNAs and genes interacting with miR-301a-3p through ncRNA-seq and RNA-seq analysis. Note: (**A**) Heatmap of DECs in mouse brain tissues from Normal (*n* = 3) and Uremia (*n* = 3) groups, with red representing upregulated genes and blue representing downregulated genes; (**B**) Heatmap of DEGs in mouse brain tissues from Normal (*n* = 3) and Uremia (*n* = 3) groups, with red representing upregulated genes and blue representing downregulated genes; (**C**) Venn diagram analysis of circRNAs predicted to interact with miR-301a-3p by ERCOI and DECs in brain tissues; (**D**) Venn diagram analysis of genes predicted to interact with miR-301a-3p by miRmap and Tarbase and DEGs in brain tissues; (**E**) Network diagram of 13 DEGs obtained from Venn analysis; (**F**) Bubble plot of GO and KEGG enrichment analysis of the 13 DEGs obtained from Venn analysis; (**G**) Network diagram of DGEs enriched in GO analysis; (**H**) Network diagram of DEGs enriched in KEGG analysis; (*n* = 3 for each group)

To explore the regulatory role of miR-301a-3p in BBB damage in UE, we conducted ncRNA-seq and transcriptome sequencing (RNA-seq) on brain tissues obtained from mice with UE. The brain tissue obtained 252 differentially expressed circRNAs (DECs, referred to as brain-circRNAs), consisting of 130 downregulated circRNAs and 122 upregulated circRNAs. These circRNAs met the condition of |logFc|> 1 and *P* < 0.05 (Fig. 3A, Table S6). After conditioning on |logFc|> 1 and *P* < 0.01, we identified 183 DEGs in brain tissue, which were referred to as brain DEGs. This set included 104 downregulated and 79 upregulated genes (Fig. 3B).

We performed an enrichment analysis of 183 DEGs using GO and the KEGG to investigate their enrichment in terms of molecular functions (MF), cellular components (CC), biological processes (BP), and signaling pathways. The results indicated that these DEGs were primarily enriched in regulating biopolymer synthesis and translation. They were also implicated in neuronal apoptosis and signaling pathways associated with neurodegeneration, Parkinson’s disease, and neurotrophic factors (Fig. S2A-B, Table S7).

This study aims to identify the circRNA sponge that interacts with miR-301a-3p in UE. We initially used the ERCOI website to predict circRNAs that target miR-301a-3p, which we called target circRNAs. Only 5 circRNAs had matching genes on the website, as shown in Figure S6. A Venn analysis was performed on target circRNAs and brain circRNAs, resulting in the identification of the DEC circRNA-PTPN4, which targets miR-301a-3p in the brain tissue of mice with UE (Fig. 3C).

This study aims to identify the specific genes (mRNA) targeted by miR-301a-3p in individuals with UE. We obtained the target genes of miR-301a-3p from the miRmap and Tarbase websites, referred to as miRmap-genes and Tarbase-genes, respectively. Next, the brain-DEGs were compared with miRmap-genes and Tarbase-genes to identify 13 genes targeted by miR-301a-3p in UE (Fig. 3D-E). Among these genes, eight showed upregulation in expression, while five showed downregulation. Figure S2C displays the PPI network of 13 DEGs.

To comprehend the enrichment of these 13 genes in the pathogenesis of UE, we carried out GO and KEGG analyses. These 13 genes demonstrated enrichment solely in response to amyloid-like proteins, regulation of neuronal apoptosis, and AMPK signaling pathway entries (Fig. 3F). Interestingly, the transcription factor, FOXO3, is consistently enriched in all entries (Fig. 3G-H; Figure S2D). These findings suggest that the transcription factor FOXO3 may function as a target gene for miR-301a-3p, which is involved in BBB injury.

**Fig. 4:**
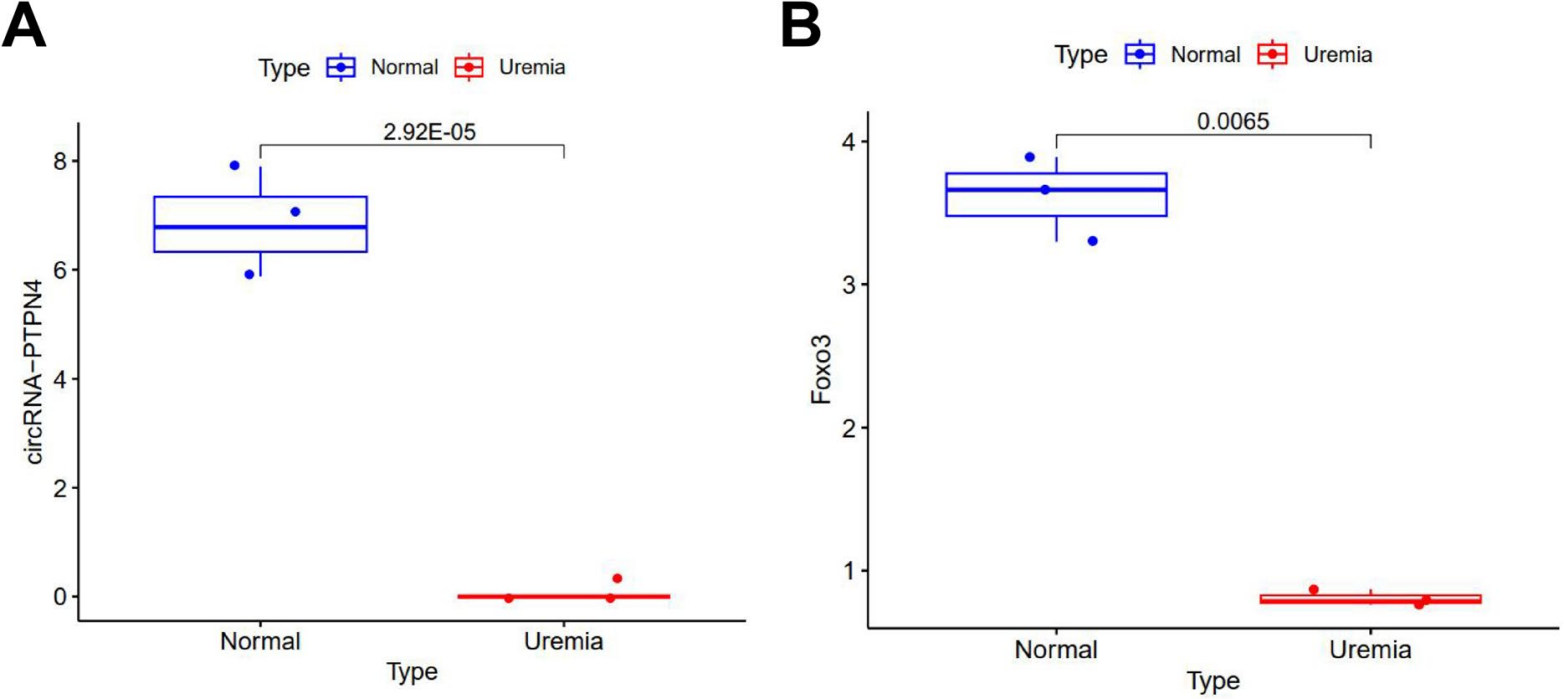
Low expression of circRNA-PTPN4 and FOXO3 in mice with UE. Note: (**A**) Expression levels of circRNA-PTPN4 in mouse brain tissues from Normal group (*n* = 3) and Uremia group (*n* = 3) as determined by ncRNA-seq; (**B**) Expression levels of FOXO3 in mouse brain tissues from Normal group (*n* = 3) and Uremia group (*n* = 3) as determined by RNA-seq; (*n* = 3 for each group)

Finally, we quantified the expression levels of circRNA-PTPN4 and FOXO3 in the brain tissue of mice with UE. The results demonstrated a downregulation in the expression levels of circRNA-PTPN4 and FOXO3 in mice with UE (Fig. 4A-B).

The results above indicate that circRNA-PTPN4 could potentially modulate the expression of FOXO3, leading to the amelioration of BBB impairment in UE through miR-301a-3p sequestration.

### Validation of the circRNA-PTPN4/miR-301a-3p/FOXO3 regulatory axis in HBMECls

**Fig. 5:**
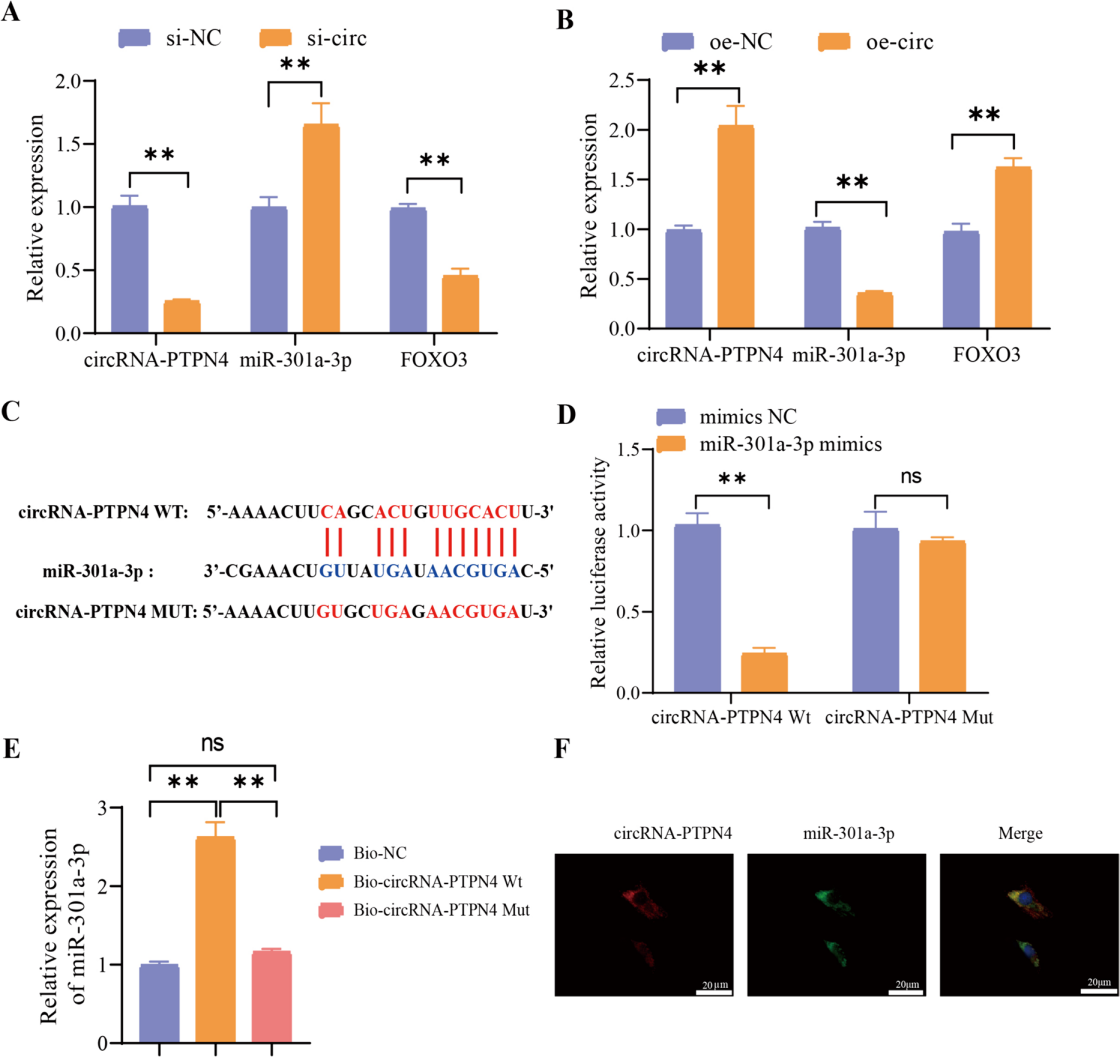
Validation of the interaction between circRNA-PTPN4 and miR-301a-3p. Note: (**A**) Influence of circRNA-PTPN4 knockdown on the expression of circRNA-PTPN4, miR-301a-3p, and FOXO3 mRNA; (**B**) Influence of circRNA-PTPN4 overexpression on the expression of circRNA-PTPN4, miR-301a-3p, and FOXO3 mRNA; (**C**) Prediction and mutation sequence of the binding sites between circRNA-PTPN4 and miR-301a-3p as predicted by ERCOI; (**D**) Verification of the predicted binding sites between circRNA-PTPN4 and miR-301a-3p using luciferase reporter gene experiment; (**E**) Verification of the binding sites between circRNA-PTPN4 and miR-301a-3p using RNA pull-down experiment; (**F**) Localization of circRNA-PTPN4 and miR-301a-3p in cells observed using FISH (scale bar = 20 μm); ns: not significant (*P* > 0.05); **: (*P* < 0.01); all cellular experiments were performed in triplicate

To validate the regulatory relationship between circRNA-PTPN4 and miR-301a-3p, stable transfectants of HBMECs with knockdown of circRNA-PTPN4 (referred to as si-circ) were established. Based on RT-qPCR experiments, neither si-circ-1 nor si-circ-2 affected the expression levels of the parental gene PTPN4 in the cells. Moreover, si-circ-1 demonstrated the most effective silencing effect. Consequently, si-circ-1 (si-circ) was used in subsequent experiments (Figure S3A). The expression level of miR-301a-3p was upregulated, whereas the mRNA expression level of FOXO3 was downregulated following the silencing of circRNA-PTPN4 (Fig. 5A). Subsequently, a stable cell line overexpressing circRNA-PTPN4 was established. The experimental validation results demonstrated successful overexpression of circRNA-PTPN4, which did not impact the expression level of the parental gene PTPN4 within the cells (Fig. S3B). Following the overexpression of circRNA-PTPN4, the expression level of miR-301a-3p was diminished, while the mRNA expression level of FOXO3 was increased (Fig. 5B).

Subsequently, we utilized the ERCOI website to predict the binding sites between circRNA-PTPN4 and miR-301a-3p. Furthermore, we generated the mutated site sequence of circRNA-PTPN4, as illustrated in Fig. 5C. Before experimenting, we validated the overexpression effect of miR-301a-3p mimics (Figure S3C). The luciferase activity assay pointed out a reduction in luciferase activity in the miR-301a-3p mimics group compared to the mimics NC group, while circRNA-PTPN4 Mut was not affected (Fig. 5D). The RNA pull-down experiment demonstrated an increase in the enrichment of miR-301a-3p in the Bio-circRNA-PTPN4 Wt group compared to the Bio-NC group.

In contrast, no change was observed in the Bio-circRNA-PTPN4 Mut group (Fig. 5E). Additionally, the FISH experiments demonstrated the co-localization of circRNA-PTPN4 and miR-301a-33p in the cytoplasm of cells (Fig. 5F). The results above indicate that circRNA-PTPN4 and miR-301a-3p co-localize in the cytoplasm of cells and directly interact with each other.

**Fig. 6:**
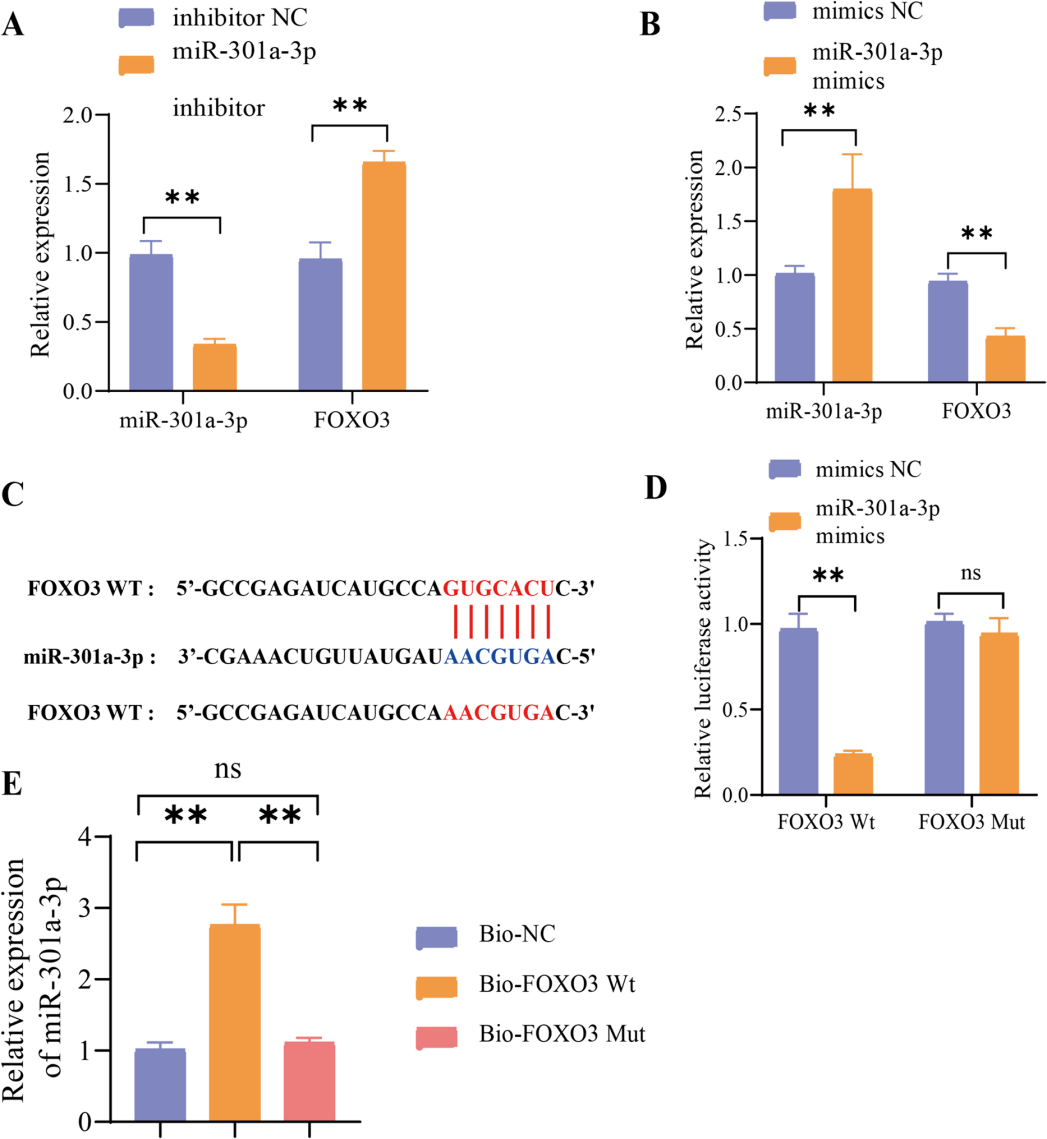
Validation of the interaction between miR-301a-3p and FOXO3. Note: (**A**) The effect of miR-301a-3p inhibitor treatment on the mRNA expression of miR-301a-3p and FOXO3; (**B**) The effect of miR-301a-3p mimics treatment on the mRNA expression of miR-301a-3p and FOXO3; (**C**) Sequence diagram illustrating the predicted binding sites between miR-301a-3p and FOXO3, as well as the mutated sites in FOXO3, predicted by miRmap website; (**D**) Confirmation of the predicted binding site between miR-301a-3p and FOXO3 through luciferase reporter gene experiment; (**E**) Verification of the predicted binding site between miR-301a-3p and FOXO3 through RNA pull-down assay; ns: not significant (*P* > 0.05); **: (*P* < 0.01); All cell experiments were repeated three times

The effectiveness of the miR-301a-3p inhibitors was verified before the experiment, as depicted in Figure S3D. Compared to the NC group of inhibitors, the miR-301a-3p inhibitors group showed upregulation of FOXO3 mRNA, as indicated by Fig. 6A. Next, we treated the cells with miR-301a-3p mimics. Notably, miR-301a-3p mimics treatment resulted in downregulating FOXO3 mRNA compared to the NC group in Fig. 6B. We subsequently used the miRmap website to predict the binding site between miR-301a-33p and FOXO3 and then created a mutant sequence of FOXO3 (Fig. 6C). The experimental results of the dual-luciferase reporter gene displayed a reduction in luciferase activity of the FOXO3 wild-type group after treatment with miR-301a-3p mimics, in comparison to the mimics NC group. However, no alteration was observed in the FOXO3 mutant group (Fig. 6D). The RNA pull-down experiment revealed an increase in the binding of miR-301a-3p to the Bio-FOXO3 Wt group compared to the Bio-NC group. However, no change was observed in the Bio-FOXO3 Mut group (Fig. 6E). Therefore, miR-301a-3p can target and inhibit the expression of FOXO3. Our assays suggest that circRNA-PTPN4 enhances the expression of FOXO3 by binding to miR-301a-3p.

### Impact of the circRNA-PTPN4/miR-301a-3p/FOXO3 axis on BBB integrity and endothelial cell function in HBMECls

**Fig. 7:**
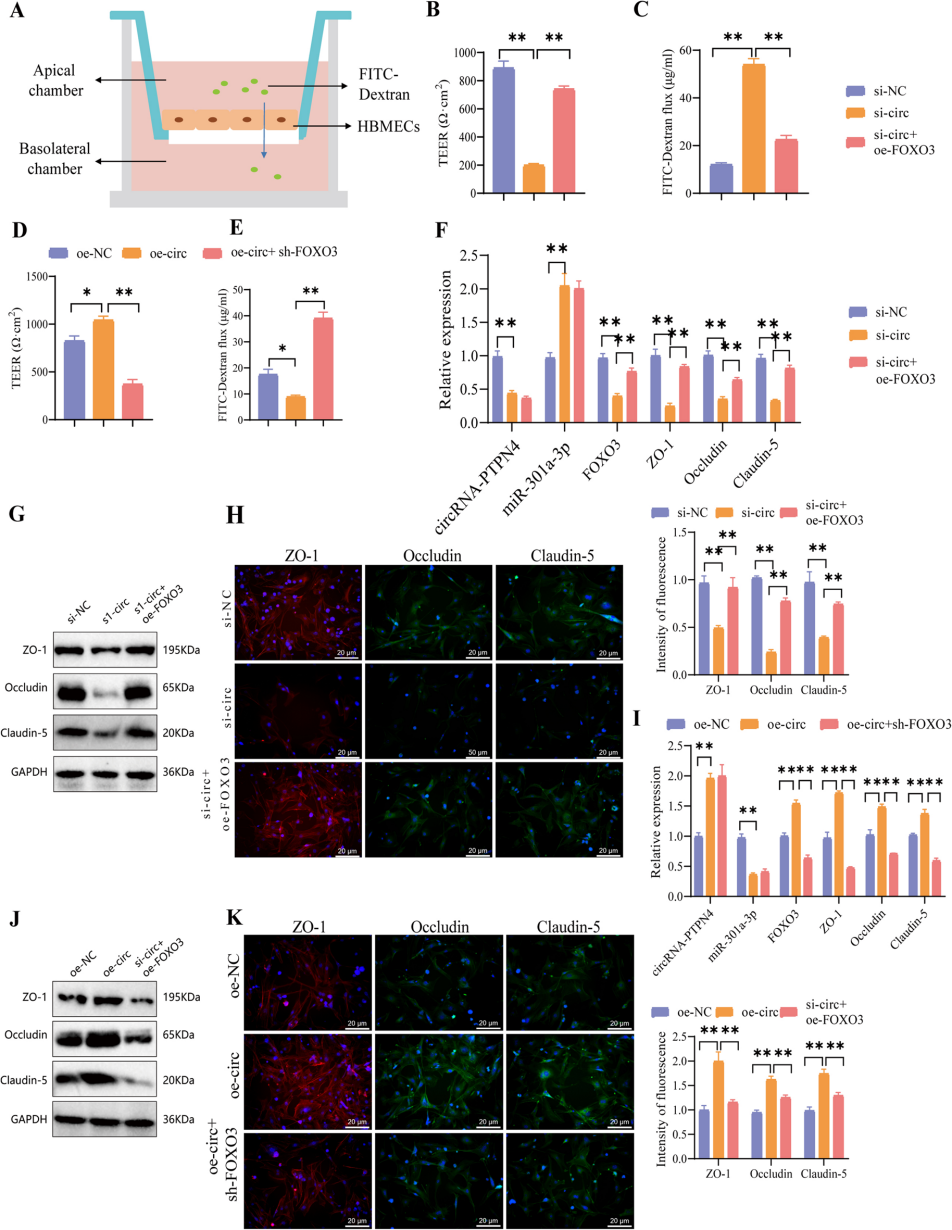
Effects of circRNA-PTPN4/miR-301a-3p/FOXO3 on the TJs of HBMECs. Note: (**A**) Schematic representation of an in vitro small chamber permeability model; (**B**) Changes in the TEER of cells after silencing circRNA-PTPN4 and overexpressing FOXO3; (**C**) Permeability of FITC-BSA in cells after silencing circRNA-PTPN4 and overexpressing FOXO3; (**D**) Changes in the TEER of cells after overexpressing circRNA-PTPN4 and silencing FOXO3; (**E**) Permeability of FITC-BSA in cells after overexpressing circRNA-PTPN4 and silencing FOXO3; (**F**) Changes in the expression of related mRNAs in cells after silencing circRNA-PTPN4 and overexpressing FOXO3; (**G**) Changes in the expression of related proteins in cells after silencing circRNA-PTPN4 and overexpressing FOXO3; (**H)** Fluorescent staining images of ZO-1, Occludin, and Claudin-5 in cells after silencing circRNA-PTPN4 and overexpressing FOXO3 (scale bar = 20 μm); (**I**) Changes in the expression of related mRNAs in cells after overexpressing circRNA-PTPN4 and silencing FOXO3; (**J**) Changes in the expression of related proteins in cells after overexpressing circRNA-PTPN4 and silencing FOXO3; (**K**) Fluorescent staining images of ZO-1, Occludin, and Claudin-5 in cells after overexpressing circRNA-PTPN4 and silencing FOXO3 (scale bar = 20 μm); ns: not significant (*P* > 0.05); *: (*P* < 0.05); **: (*P* < 0.01); All cell experiments were repeated three times

Further investigation is needed to examine the effects of the circRNA-PTPN4/miR-301a-3p/FOXO3 axis on the integrity of the BBB. Initially, the cellular TEER was determined using a TEER analyzer that adheres to the small room transparency model. A FITC-Dextran permeability experiment was also conducted to evaluate cell permeability (Fig. 7A). Following the silencing of circRNA-PTPN4, we detected a reduction in TEER and an elevation in fluorescence intensity, suggesting increased permeability and enhanced leakage of large molecules into the basolateral chamber. When circRNA-PTPN4 was knocked down, and FOXO3 was overexpressed, the recovery of TEER and the decrease in fluorescence intensity of large molecular permeation into the basolateral chamber indicated a decrease in cellular permeability (Fig. 7B-C). When circRNA-PTPN4 is overexpressed, the TEER increased, while the permeability of fluorescence-labeled macromolecules decreased. However, the opposite results are observed when this overexpression is combined with FOXO3 silencing (Fig. 7D-E). (The silencing and overexpression effects of FOXO3 were verified before the experiment, as depicted in Figure S4A-D). Considering sh-FOXO3-1 exhibited the most effective silencing, it was selected for subsequent experiments and referred to as sh-FOXO3).

Tight junction (TJ) structures consist of various proteins, primarily transmembrane proteins like Occludin and Claudin-5, and peripheral membrane proteins like ZO-1. Specifically, the ZO-1 protein serves as a crucial connector between the extracellular TJs and intracellular actin, playing a pivotal role in preserving the structural integrity of TJs (Haas et al. 2022, 2020).

We observed that silencing circRNA-PTPN4 resulted in an upregulation of miR-301a-3p expression, while the mRNA and protein levels of FOXO3 and ZO-1 were downregulated. Moreover, the expression of other TJ proteins, such as Occludin and Claudin-5, was also reduced. Notably, the overexpression of FOXO3 counteracted the downregulation of these proteins, as demonstrated in Fig. 7F-G. Immunofluorescence analysis revealed a notable reduction in fluorescence signals of ZO-1, Occludin, and Claudin-5 in circRNA-PTPN4-silenced cells, accompanied by evident signs of disruption and loss. Overexpression of FOXO3 could enhance the fluorescent signal of these proteins (Fig. 7H). Overexpression of circRNA-PTPN4 leads to downregulation of miR-301a-3p expression and enhancement of ZO-1 expression levels and intercellular signaling. Conversely, silencing FOXO3 produces opposite results (Fig. 7I-K).

**Fig. 8:**
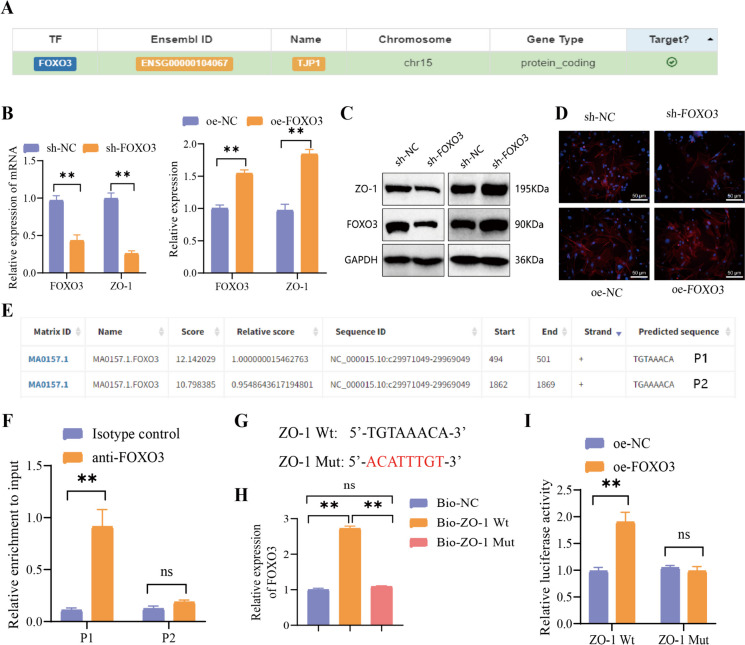
Regulation of ZO-1 transcription by FOXO3 as a transcription factor. Note: (**A**) Prediction of FOXO3 as a transcription factor for ZO-1 using the hTFtarget website; (**B**) Changes in the expression of related mRNAs after silencing FOXO3; (C) Changes in the expression of related proteins after silencing FOXO3; (**D**) Fluorescent staining images of ZO-1 in cells after silencing FOXO3 (scale bar = 20 μm); (**E)** Prediction of binding sites between FOXO3 and ZO-1 obtained from the JASPAR website (https://jaspar.genereg.net/); (**F**) Verification of potential binding sites between FOXO3 and ZO-1 through ChIP-PCR experiment; (**G**) Sequence diagram illustrating the binding sites between FOXO3 and ZO-1, as well as the mutated sites in ZO-1; (**H**) Confirmation of the binding sites between FOXO3 and ZO-1 through DNA pull-down assay; (**I**) Verification of the binding sites between FOXO3 and ZO-1 through luciferase reporter gene experiment; ns: not significant (*P* > 0.05); **: (*P* < 0.01); All cell experiments were repeated three times

Interestingly, a transcriptional regulatory relationship between FOXO3 and ZO-1 was unveiled, which was obtained from the hTFtarget website (Fig. 8A). Further experiments based on RT-qPCR, Western blot, and immunofluorescence uncovered a reduction in mRNA and protein levels, consequently disrupting intercellular signaling. In contrast, overexpression of FOXO3 yielded the opposite outcome (Fig. 8B-D). Hence, we hypothesize that FOXO3 acts as a transcription factor for ZO-1, controlling its expression and influencing the integrity of TJ structures. To confirm the hypothesis, we utilized the JASPAR website to predict two potential binding sites with high scores between FOXO3 and ZO-1 (Fig. 8E). Subsequently, experimental validation of these predicted binding sites was performed. The experimental results from the ChIP-PCR demonstrated the enrichment of FOXO3 at the P1 sequence (Fig. 8F). The primary sequence connecting FOXO3 and ZO-1 is the P1 sequence. In subsequent experimental designs, mutated versions of the P1 sequence were used, labeled as ZO-1 Wt and ZO-1 Mut in Fig. 8G. Subsequently, we employed biotinylated DNA probes labeled with wild-type ZO-1 (ZO-1 Wt) and mutant ZO-1 (ZO-1 Mut) to conduct DNA pull-down experiments to further substantiate the binding of site 1. The results demonstrate a specific interaction between FOXO3 and ZO-1 Wt. However, no specific interaction was observed between FOXO3 and ZO-1 Mut (Fig. 8H). The luciferase activity assay results indicated that the oe-FOXO3 group exhibited higher fluorescence enzyme activity of ZO-1 Wt compared to the oe-NCs group. Conversely, the ZO-1 Mut group showed no change in activity (Fig. 8I). The above findings suggest that FOXO3 can enhance the transcription of ZO-1 and modulate the assembly of TJs.

**Fig. 9:**
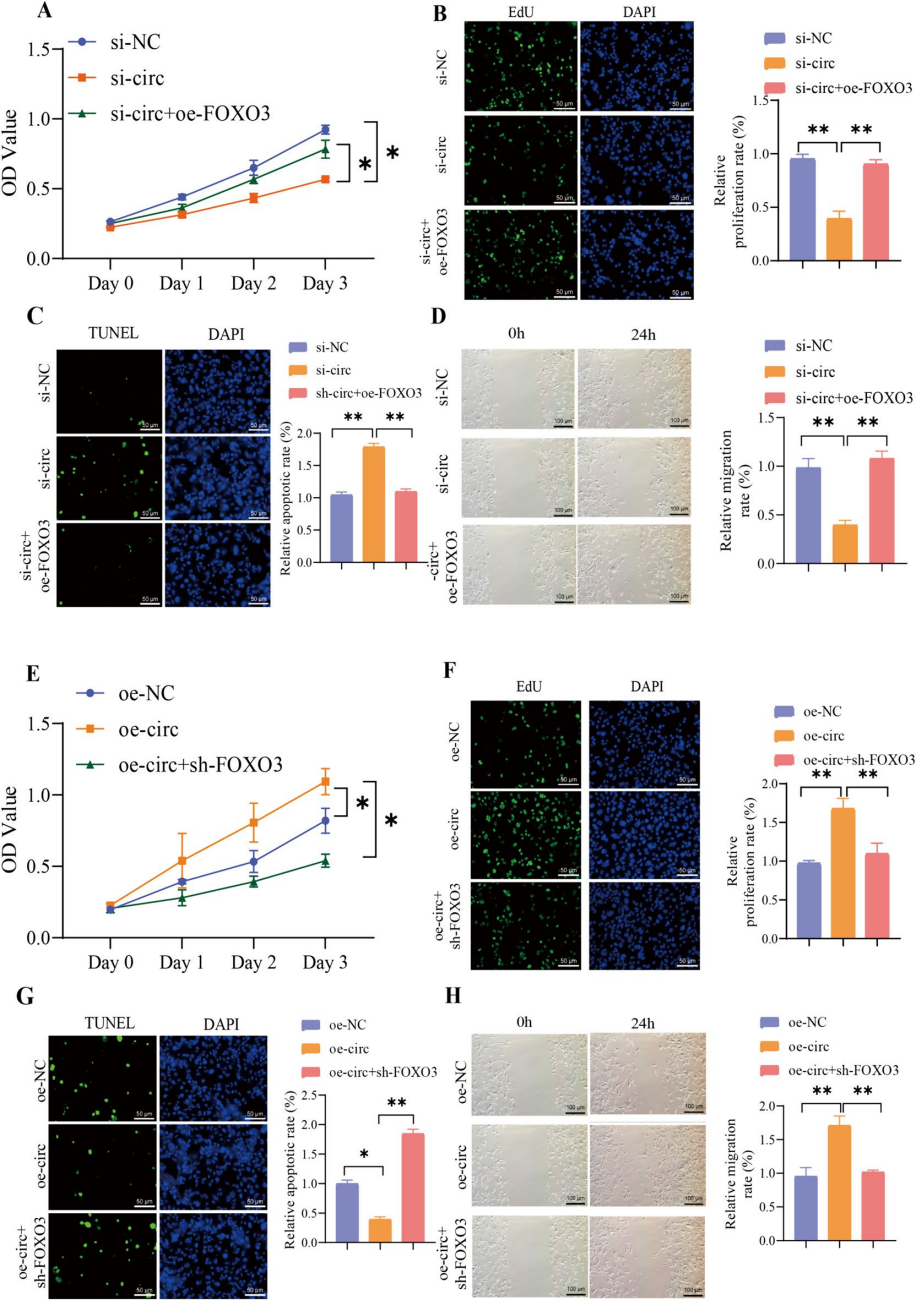
Effects of circRNA-PTPN4/miR-301a-3p/FOXO3 on the growth, proliferation, and migration of HBMECs. Note: (**A**) The effect of silencing circRNA-PTPN4 and overexpressing FOXO3 on cell growth activity; (**B**) The effect of silencing circRNA-PTPN4 and overexpressing FOXO3 on cell proliferation (scale bar = 50 μm); (**C**) The effect of silencing circRNA-PTPN4 and overexpressing FOXO3 on cell apoptosis (scale bar = 50 μm); (D) The effect of silencing circRNA-PTPN4 and overexpressing FOXO3 on cell migration (scale bar = 100 μm); (**E**) The effect of overexpressing circRNA-PTPN4 and silencing FOXO3 on cell growth activity; (**F**) The effect of overexpressing circRNA-PTPN4 and silencing FOXO3 on cell proliferation (scale bar = 50 μm); (**G**) The effect of overexpressing circRNA-PTPN4 and silencing FOXO3 on cell apoptosis (scale bar = 50 μm); (**H**) The effect of overexpressing circRNA-PTPN4 and silencing FOXO3 on cell migration (scale bar = 100 μm); *: (*P* < 0.05); **: (*P* < 0.01); All cell experiments were repeated three times

We further examined the alterations in distinct cell populations’ growth, proliferation, and migration capacities. Notably, the downregulation of circRNA-PTPN4 led to a decrease in cell growth, proliferation, and migration, accompanied by increased apoptosis. In contrast, the overexpression of FOXO3 had the opposite effect (Fig. 9A-D). The overexpression of circRNA-PTPN4 facilitated cell growth, proliferation, and migration while decreasing apoptosis. In contrast, the suppression of FOXO3 yields contrasting outcomes (Fig. 9E-H).

The results above demonstrate that the circRNA-PTPN4/miR-301a-3p/FOXO3 axis promotes the formation of TJs in HBMECs by regulating the transcription of ZO-1, thus influencing the functionality of the BBB.

### Modulation of BBB integrity and cognitive function by the circRNA-PTPN4/miR-301a-3p/FOXO3 axis in a mouse model of UE

**Fig. 10:**
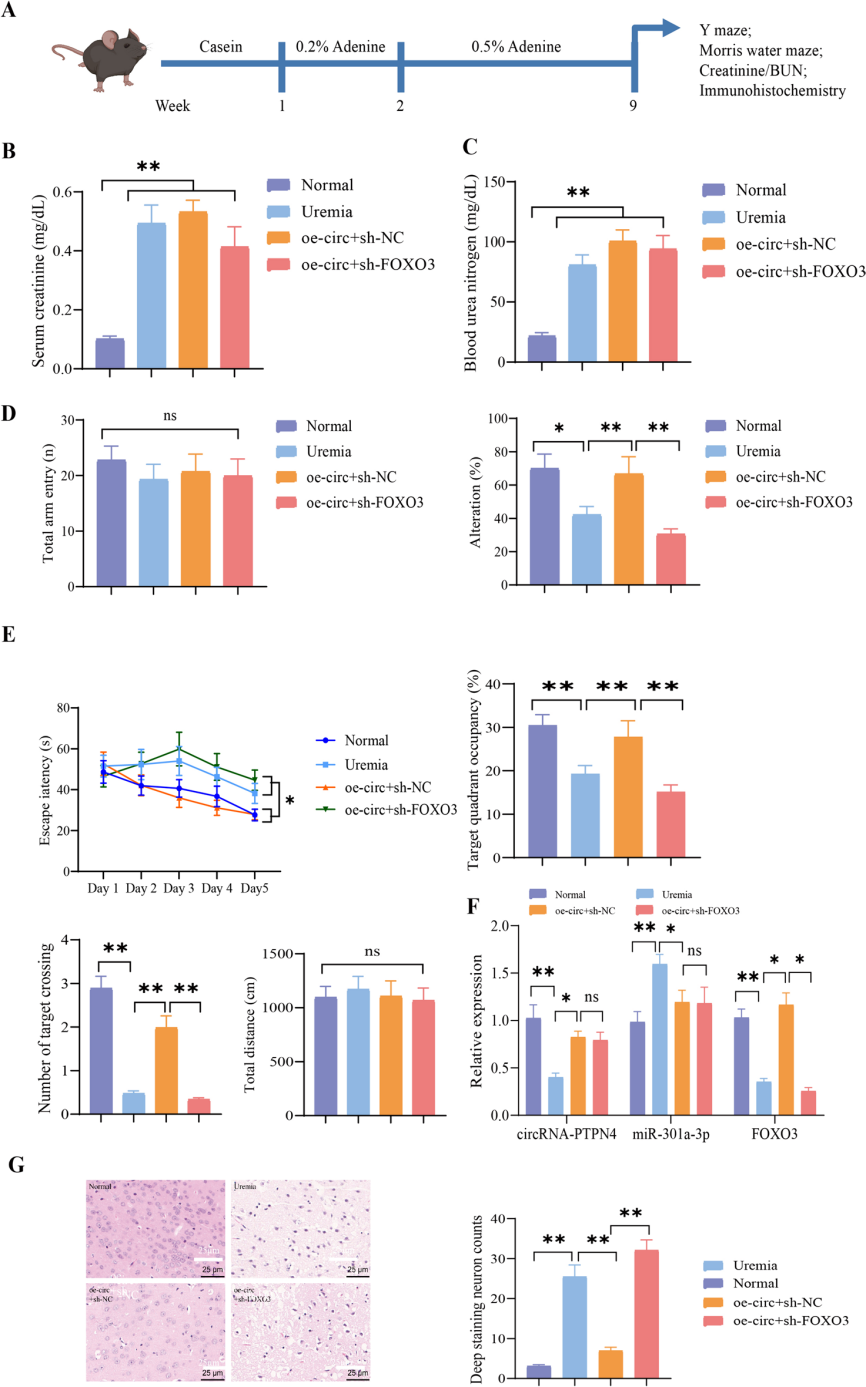
Effects of circRNA-PTPN4/miR-301a-3p/FOXO3 on cognitive function in mice with UE. Note: (**A**) Schematic representation of the model construction for mice with UE; (**B**) Measurement of creatinine levels in mouse blood; (**C**) Measurement of blood urea nitrogen levels in mice; (**D**) Statistical analysis of total arm entries and percentage alternation in the Y-maze test of mice; (**E**) Statistical analysis of escape latency, target quadrant occupancy, number of target crossings, and total traveled distance in the Morris water maze test of mice; (**F**) Changes in the mRNA expression of circRNA-PTPN4, miR-301a-3p, and FOXO3 in mice from each group; (**G**) HE staining results of brain tissue slices from mice in each group (scale bar = 25 μm); (*n* = 15 for each group); ns: not significant (*P* > 0.05); *: (*P* < 0.05); **: (*P* < 0.01)

We created a UE mouse model to investigate the impact of the circRNA-PTPN4/miR-301a-3p/FOXO3 axis on the BBB. This model involved lentivirus infection in the cerebral ventricles to simultaneously overexpress circRNA-PTPN4 and silence FOXO3 (Fig. 10A). To validate the successful establishment of the model, we performed tests on indicators related to uremia. The results demonstrated elevated creatinine and blood urea nitrogen (BUN) levels in mice with UE compared with normal mice. These findings suggest renal impairment in the model mice (Fig. 10B-C).

Subsequently, we evaluated the cognitive function of mice using the Y-maze test and the Morris water maze test. The results indicated a reduction in the percentage of transitions in the Y-maze test within the mice with the UE group. However, no statistical difference was observed in total arm entries (Fig. 10D). In the Morris water maze experiment, the UE group of mice showed increased escape latency compared to the control group. Additionally, the occupancy rate and the number of target crossings were diminished. However, there was no difference in the total distance traveled. These findings suggest a decline in cognitive function in mice (Fig. 10E). The cognitive function was enhanced in mice from the oe-circ + sh-NC group, whereas cognitive function was diminished in mice following the silencing of FOXO3 (Fig. 10D-E).

Following that, we isolated brain tissues from mice. The findings of the RT-qPCR experiment revealed downregulation in the expression levels of circRNA-PTPN4 and FOXO3 mRNA in mice with UE, while the expression level of miR-301a-3p was upregulated. Overexpression of circRNA-PTPN4 resulted in the downregulation of miR-301a-3p expression while upregulating the expression of FOXO3. Conversely, silencing FOXO3 led to the downregulation of circRNA-PTPN4 expression (Fig. 10F). These findings suggest that circRNA-PTPN4/miR-301a-3p modulates FOXO3 to impact cognitive function in mice with UE.

**Fig. 11:**
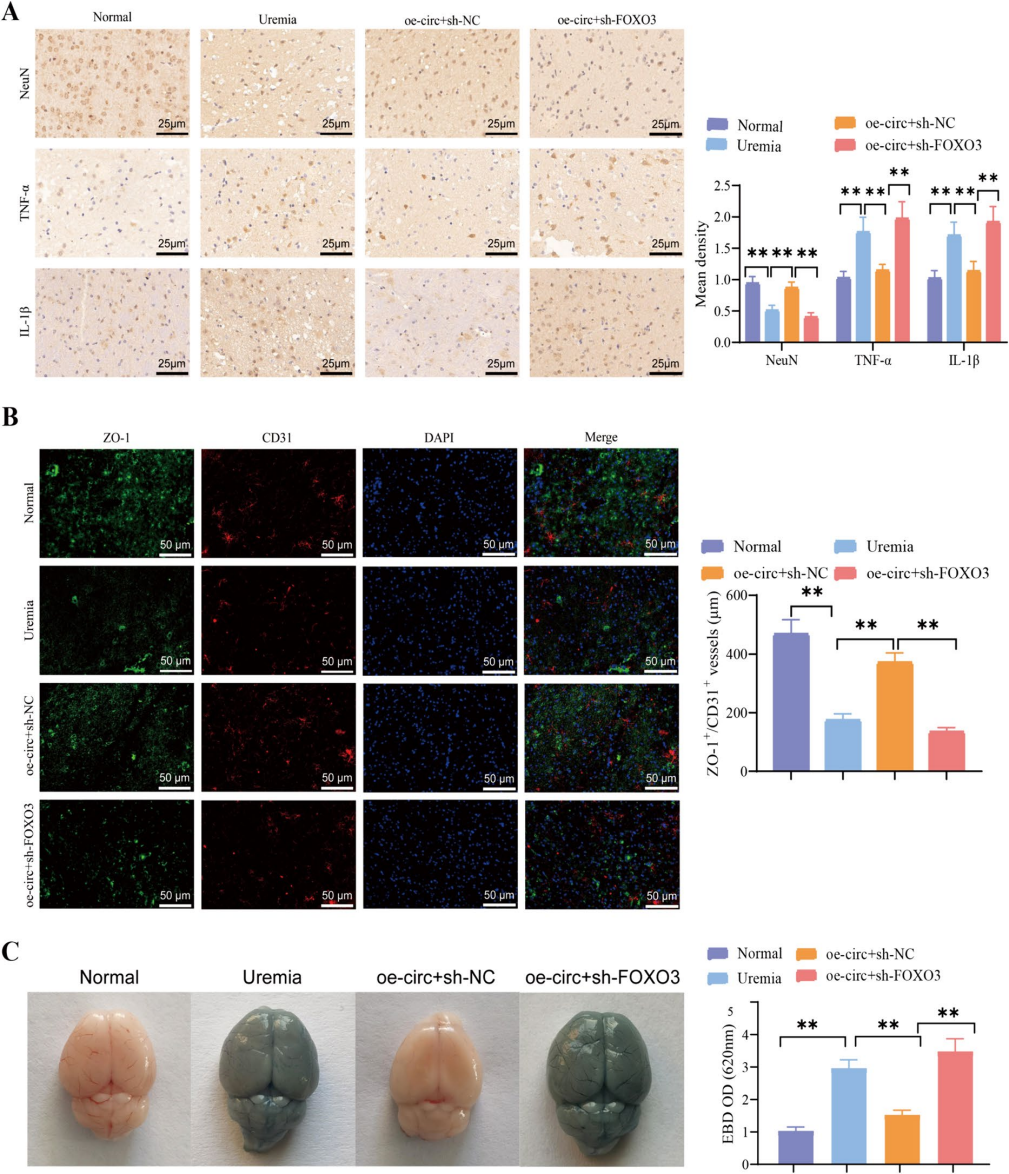
Influence of circRNA-PTPN4/miR-301a-3p/FOXO3 axis on BBB in mice with UE. Note: (**A**) Immunohistochemical staining of neuronal marker NeuN and inflammatory markers TNF-α, IL-1β in brain tissue sections of mice in different groups (scale = 25 μm). (**B**) Immunohistochemical staining of TJ protein ZO-1 and vascular marker CD31 in brain tissue sections of mice in different groups (scale = 50 μm). (**C**) Evaluation of BBB permeability in mouse brain tissue using Evans Blue staining. (*n* = 15 in each group). **: (*P* < 0.01)

Moreover, we performed a pathological analysis and conducted immunohistochemical staining on mouse brain tissue. The H&E staining revealed pronounced cytoplasmic atrophy and neuronal abnormalities in the brains of the mice in the UE model group. The cytoplasm of cells and neurons in the oe-circ + sh-NC group mice returned to normal, while the cytoplasm of cells in the oe-circ + sh-FOXO3 group mice shrank further and neurons displayed abnormalities (Fig. 10G). Immunohistochemical staining results of the immune organs revealed a reduction in the NeuN levels, a neuronal marker, in the brains of mice with UE. This reduction indicates the occurrence of neuronal loss in brain tissues. The levels of TNF-α, IL-1β, and other neuroinflammatory biomarkers increased, suggesting the existence of inflammation infiltration in the brain tissue (Fig. 11A). In the mice of the group treated with oe-circ + sh-NC, the observed pathological changes and degree of inflammation showed improvement. However, when FOXO3 was silenced, the pathological changes and inflammation worsened (Fig. 11A). Subsequently, brain tissue blood vessels were labeled with CD31 using immunofluorescence staining, and the expression of ZO-1 was assessed. The results indicated a reduction in ZO-1 staining in the brains of mice in the UE group compared to the normal group. Following the overexpression of circRNA-PTPN4, the expression of ZO-1 is reinstated, whereas the downregulation of FOXO3 has the converse effect (Fig. 11B).

Finally, we assessed the permeability of the BBB in mice by employing Evans Blue staining. The findings demonstrated that mice with UE and the oe-circ + sh-FOXO3 group displayed considerable extravasation of Evans blue, signifying a substantial elevation in the permeability of the BBB. In the circRNA-PTPN4 overexpression group of mice, BBB permeability diminished (Fig. 11C).

In conclusion, circRNA-PTPN4 promotes the expression of FOXO3 and upregulates ZO-1 by absorbing miR-301a-3p. This restoration of the BBB function in mice with UE subsequently improves their cognitive abilities.

## Discussion

UE is a neurological complication associated with uremia, yet its pathogenesis remains incompletely understood (Rosner et al. 2022). In recent years, there has been extensive research on the role of ncRNA in the pathogenesis of various diseases. However, its role in UE remains unclear (Zhang et al. 2020; Yan and Bu 2021). This study uncovers the crucial role of the circRNA-PTPN4/miR-301a-3p/FOXO3 axis in UE using high-throughput sequencing of ncRNA. This groundbreaking discovery complements existing research and opens new avenues for further disease investigation.

Dysfunction of the BBB has been implicated in the pathogenesis of a range of neurological disorders, underscoring its critical role as a protective interface between the brain and external milieu (Kadry et al. 2020). Prior research has posited a potential link between UE and compromised BBB integrity, yet the underlying biological mechanisms have yet to be fully elucidated (Sina et al. 2022). In this investigation, high-throughput sequencing techniques were employed to reveal an elevated expression of miR-301a-3p in mice with UE (Figs. 1 and 2). Subsequent application of ncRNA-seq and RNA-seq methodologies, alongside predictive analyses from various databases, identified circRNA-PTPN4 as a DEC targeting miR-301a-3p within the cerebral tissues of these mice (Fig. 3). The regulatory interaction among circRNA-PTPN4, miR-301a-3p, and FOXO3 was validated through a series of experiments, including circRNA-PTPN4 silencing and overexpression, dual-luciferase reporter assays, RNA pull-down assays, fluorescent in situ hybridization, and treatments with miR-301a-3p inhibitors (Figs. 5 and 6), indicating a direct interaction and co-localization between circRNA-PTPN4 and miR-301a-3p, thereby facilitating the expression of FOXO3. Further analyses elucidated the impact of the circRNA-PTPN4/miR-301a-3p/FOXO3 axis on the expression and structural integrity of tight junction proteins (Figs. 7–9). ZO-1, a pivotal protein in the maintenance of tight junction integrity and thus BBB functionality, acts as a critical bridging molecule between cells, essential for the coherence of cellular junctions in HBMECs (Haas et al. 2022, 2020). The establishment of a mouse model of UE through intracerebroventricular administration of lentivirus for simultaneous overexpression of circRNA-PTPN4 and silencing of FOXO3 demonstrated that the in vivo circRNA-PTPN4/miR-301a-3p/FOXO3 regulatory axis could ameliorate BBB dysfunction and restore cognitive abilities in mice (Figs. 10 and 11). This investigation substantiates the notion that the circRNA-PTPN4/miR-301a-3p/FOXO3 axis plays a crucial role in modulating BBB function by regulating the expression of ZO-1, thus offering new insights into the mechanistic involvement of ncRNAs, particularly circRNAs, in cellular signal transduction and providing a novel model for understanding the complex dynamics of BBB integrity and neurological diseases.

An increasing body of research is focusing on the role of ncRNAs in the etiology and mechanisms of action across various diseases. Studies have identified miR-370-3p in the brain and plasma as a potential biomarker for sepsis-associated encephalopathy (SAE), though not for UE (Visitchanakun et al. 2020). Similarly, the levels of miR-146a in the brain have been shown to assist in the diagnosis, prognosis, or treatment of transmissible spongiform encephalopathies (TSEs) (Pogue et al. 2022), while miR-30b can modulate the disease course and immune response in hypoxic-ischemic encephalopathy (HIE) through the regulation of PAI-1 (Wang and Jia 2021). These findings underscore the significant advantages of circRNAs and miRNAs as molecular markers in the diagnosis of neurological diseases. Moreover, there is a growing interest in the therapeutic potential of the circRNA-miRNA-mRNA regulatory network. In the field of oncology, He and colleagues observed an upregulation of hsa_circ_0005239 in hepatocellular carcinoma tissues and cell lines. Overexpression of hsa_circ_0005239 could counteract the suppression of the target gene PD-L1 by miR-34a-5p through a ceRNA mechanism, facilitating cellular migration in hepatocellular carcinoma (He et al. 2023). In the context of neurological injury, research by Yu et al. found that circ-003423 is downregulated in an ox-LDL-induced HBMEC-IM cell model of brain vascular endothelial cell damage. Overexpressing circ-003423 alleviated ox-LDL-induced damage by competitively inhibiting miR-589-5p, thereby mitigating the suppression of TET2 expression and enhancing cellular proliferation and migration (Yu et al. 2021). Additionally, Zhou and colleagues demonstrated that circ-EPS15 is downregulated in both Parkinson’s disease (PD) patients and mouse models. Overexpression of circ-EPS15 could absorb miR-24-3p, enhancing PINK1-PRKN-mediated mitophagy, thereby ameliorating neurological damage (Zhou et al. 2023). In an oxygen–glucose deprivation (OGD)-induced HBMECs model, circ-129657 competitively bound with miR-194-5p to regulate GMFB expression. Silencing circ-129657 upregulated GMFB, promoting endothelial cell proliferation, reducing brain infarct volume, and mitigating neurological damage in MCAO mouse models (Qian et al. 2023). While circRNA-miRNA axes have been extensively explored in cancer and neurological diseases such as Alzheimer’s and Parkinson’s, research on their role in UE is sparse.

FOXO3, a transcription factor recognized for its roles in cellular proliferation, programmed cell death, and oxidative stress management (Hoong and Chua 2021; McIntyre et al. 2022; Zhao and Liu 2021; Yang et al. 2021), is identified as a critical regulator of the BBB, with particular emphasis on its implications in UE. This research underscores FOXO3’s integral function and initiates avenues for examining its neuroprotective effects. While focusing predominantly on molecular dynamics, the outcomes hint at promising therapeutic prospects for managing UE. Notably, adjustments in the circRNA-PTPN4 and FOXO3 expressions emerge as potential therapeutic strategies for this disorder. The investigation provides a comprehensive analysis of ncRNA’s involvement in UE, linking it with BBB functionality (Graphical Abstract). Based on high-throughput sequencing alongside bioinformatic techniques, we delineated a key ncRNA regulatory pathway, the circRNA-PTPN4/miR-301a-3p/FOXO3 axis. Laboratory assays underscored the pathway’s relevance in modulating cellular permeability, proliferation, and migration. Particularly, circRNA-PTPN4 inhibits miR-301a-3p, facilitating an increased expression of FOXO3, a mechanism corroborated through in vivo studies. The UE mouse model illustrated that circRNA-PTPN4 overexpression could significantly enhance cognitive functions, mitigate neuronal loss and inflammation, and ameliorate BBB disturbances. Thus, circRNA-PTPN4 is posited as a protective agent in UE development, laying the groundwork for future investigations into its molecular underpinnings and therapeutic potential.

**Fig. 12:**
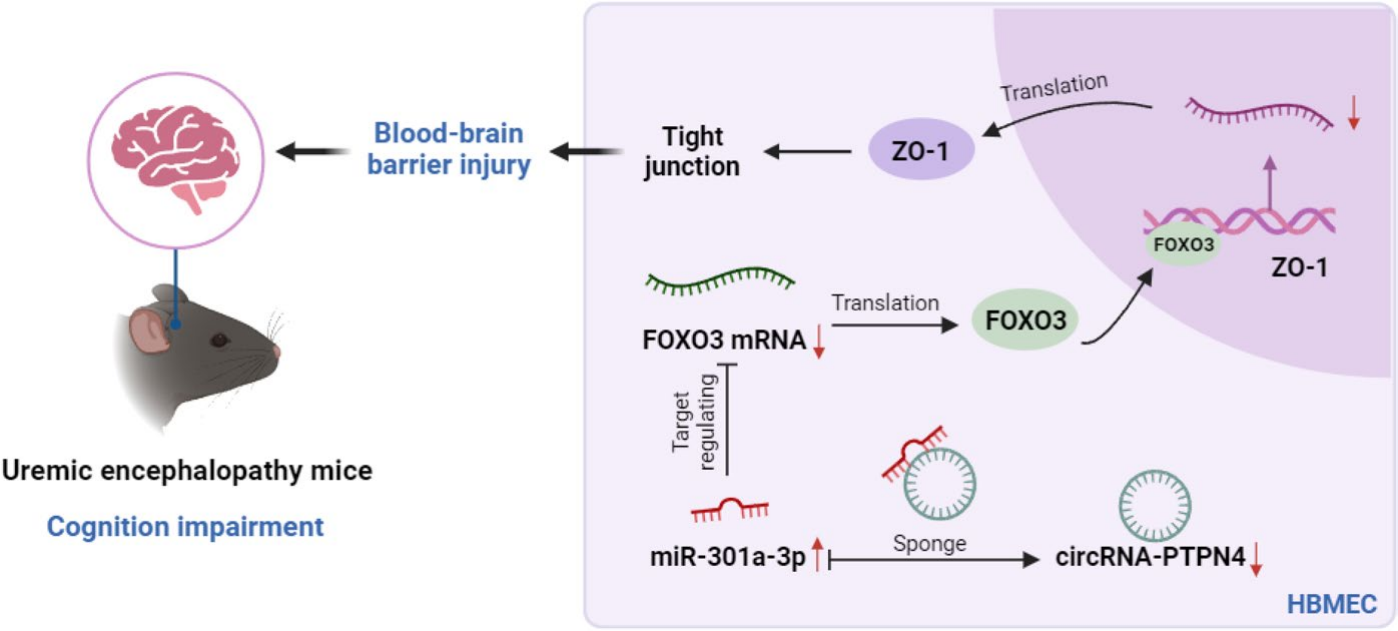
Involvement of circRNA-PTPN4/miR-301a-3p/FOXO3 axis in the occurrence and progression of UE through influencing BBB function

This research reveals the significant impact of the circRNA-PTPN4/miR-301a-3p/FOXO3 signaling axis on UE progression, providing novel insights into the complex molecular mechanisms characterizing this neurological ailment (Fig. 12). By validating the essential influence of ncRNAs, specifically circRNAs and miRNAs, in neurological diseases, the study accentuates the importance of deepening our understanding of ncRNAs in future research. The identification of the circRNA-PTPN4/miR-301a-3p/FOXO3 axis as a viable target opens avenues for innovative therapeutic interventions, promising more precise and efficacious treatment modalities for individuals afflicted with UE. Consequently, the findings introduce new molecular biomarkers for the early detection and prognostic assessment of UE, carrying significant clinical implications that could lead to timely interventions and enhanced patient outcomes.

Over the past decade, research on circRNAs has made significant strides. However, investigations within the domain of neurological diseases have predominantly focused on SAE and HIE, with a relatively unilateral approach toward exploring the mechanisms underlying UE and a scarcity of related studies. This relative void in research hampers the ability to integrate and refine existing studies thoroughly, potentially leading to subjective biases and overlooking pertinent knowledge from adjacent fields. Such limitations could impede the identification and resolution of research gaps and deficiencies, as well as the proposition of novel research directions and improvements, consequently restricting the depth and breadth of analysis. Moreover, the pathogenesis of UE is recognized as complex, likely resulting from multifactorial contributions, including alterations in hormonal metabolism, retention of uremic solutes, shifts in electrolyte and acid–base balance, blood–brain barrier transport, changes in vascular reactivity, and inflammation, without established diagnostic criteria and with variable clinical presentations (Olano et al. 2023). Although mouse models have provided valuable insights, physiological and metabolic differences between mice and humans suggest that findings might not be fully applicable to human cases. Furthermore, while this study centers on the circRNA-PTPN4/miR-301a-3p/FOXO3 axis, the network of ncRNAs is exceedingly complex. Given the broad range of miRNA downstream targets and the complexity of their interactions, other significant ncRNAs may be implicated in the pathogenesis of UE. Although some molecular mechanisms have been uncovered, other unknown pathways and mechanisms related to the progression of UE may exist. Future research needs to validate these findings in larger sample populations and further explore their relevance in humans. Current studies on the association between circRNAs and disease predominantly revolve around the circRNA-miRNA-mRNA regulatory network; however, whether this represents the primary function of circRNAs within organisms remains uncertain.

Future research directions should likely involve comprehensive analyses of circRNAs’ interactions with proteins, direct regulation of gene expression, and protein translation, leveraging bioinformatics’ big data, and drawing from research methodologies used in miRNA and lncRNA studies to establish an exhaustive and continuously updated circRNA database. This approach will integrate various ncRNA studies for a comprehensive analysis of the circRNA functional network, facilitating deeper investigations into ncRNAs, particularly circRNAs and miRNAs, in UE. This may unveil additional mechanisms and therapeutic targets. Based on this research, the development of therapeutic drugs or strategies targeting the circRNA-PTPN4/miR-301a-3p/FOXO3 axis could commence, offering improved treatment options for patients with UE. In summary, while this study provides new insights into the pathogenesis of UE, further research is necessary to validate these findings and translate them into clinical applications.

## Supplementary information

Below is the link to the electronic supplementary material.

Figure S1. Meta-analysis of miR-301a-3p subgroups (sequencing methods). Note: Forest plot comparing Normal group and BBB injury group in miR-301a-3p subgroup (sequencing methods); SMD: standard mean difference; 90% CI: 95% confidence interval. (JPG 2011 KB)

Figure S2. Analysis of ncRNA-seq and selection of genes interacting with miR-301a-3p. Note: (**A**) Bubble plot of GO analysis of DGEs in RNA-seq data. (**B**) Bubble plot of KEGG analysis of DGEs in RNA-seq data. (**C**) PPI network of the 13 DEGs obtained from Venn analysis. (**D**) Venn analysis shows enrichment of the 13 DEGs in GO and KEGG terms. (*n* = 3 in each group). (JPG 2126 KB)

Figure S3. Interaction between miR-301a-3p and FOXO3. Note: (**A**) Expression changes of circRNA-PTPN4 after circRNA-PTPN4 silencing. (**B**) Expression changes of circRNA-PTPN4 after circRNA-PTPN4 overexpression. (**C**) Expression changes of miR-301a-3p after miR-301a-3p mimics treatment. (**D**) Expression changes of miR-301a-3p after miR-301a-3p inhibitor treatment. ns: not significant (P > 0.05); *: (P < 0.05); **: (P < 0.01); All cell experiments were repeated 3 times. (JPG 568 KB)

Figure S4. Validation of the effects of FOXO3 silencing and overexpression. Note: (**A**) Expression changes of mRNA after FOXO3 silencing. (**B**) Expression changes of protein after FOXO3 silencing. (**C**) Expression changes of mRNA after FOXO3 overexpression. (**D**) Expression changes of protein after FOXO3 overexpression. *: (P < 0.05); **: (P < 0.01); All cell experiments were repeated 3 times. (JPG 515 KB)

Supplementary file 5 (DOCX 18 KB)

Supplementary file 6 (PDF 4291 KB)

## Data Availability

No datasets were generated or analysed during the current study.

## Abbreviations

cDNA: Complementary DNA
bp: Base pairs
DEGs: Differentially expressed genes
HBMEC: Human brain microvascular endothelial cells
FBS: Fetal bovine serum
Wt: Wild-Type
TEER: Transendothelial electrical resistance
H&E: Hematoxylin and eosin
OD: Optical density
ncRNA: Non-coding RNA
DEM: Differentially expressed microRNA
