# Supplementary figures and images for: circRNA-PTPN4 mediated regulation of FOXO3 and ZO-1 expression: implications for blood–brain barrier integrity and cognitive function in uremic encephalopathy
Source: Cell Biol Toxicol. 2024 Apr 17;40(1):22. doi: 10.1007/s10565-024-09865-6 (PMC11024022; doi:10.1007/s10565-024-09865-6)

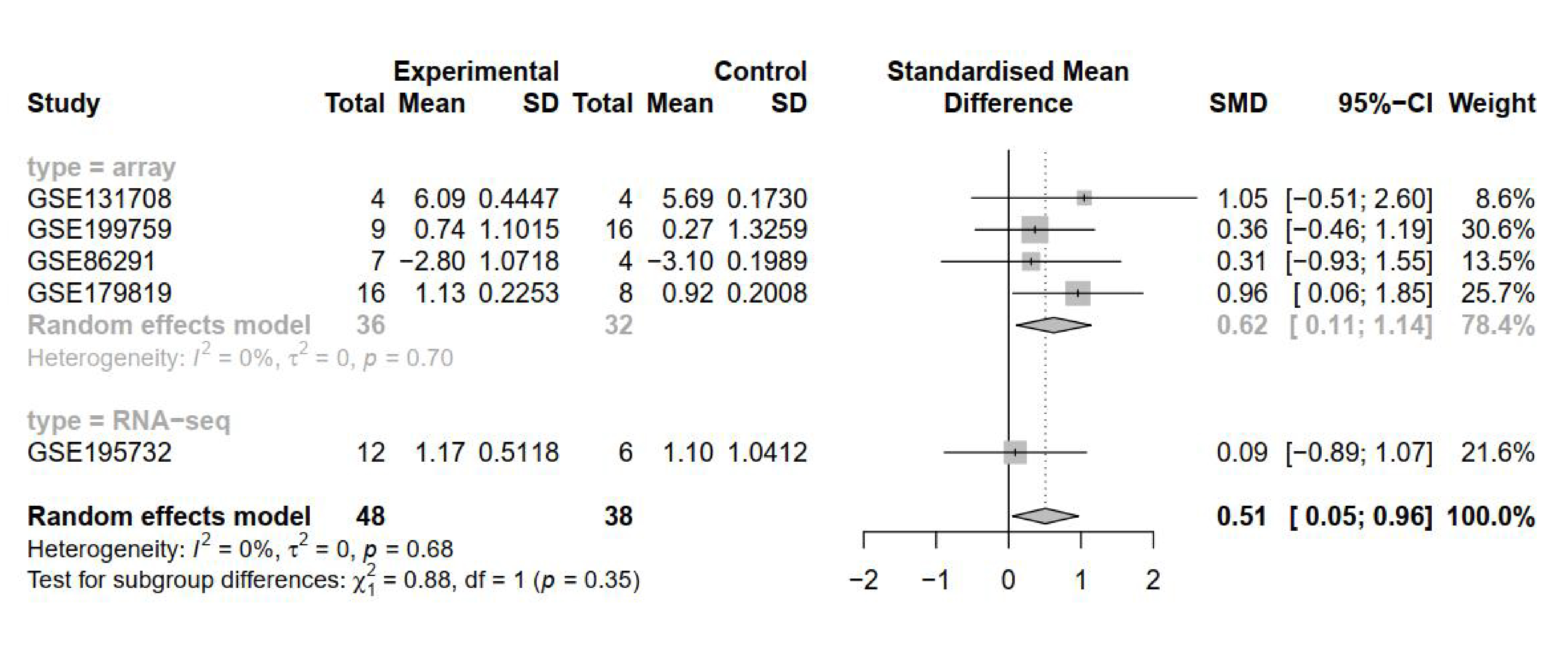

Supplement: Supplementary file 1 — Figure S1. Meta-analysis of miR-301a-3p subgroups (sequencing methods). Note: Forest plot comparing Normal group and BBB injury group in miR-301a-3p subgroup (sequencing methods); SMD: standard mean difference; 90% CI: 95% confidence interval. (JPG 2011 KB) [file 10565_2024_9865_MOESM1_ESM.jpg]

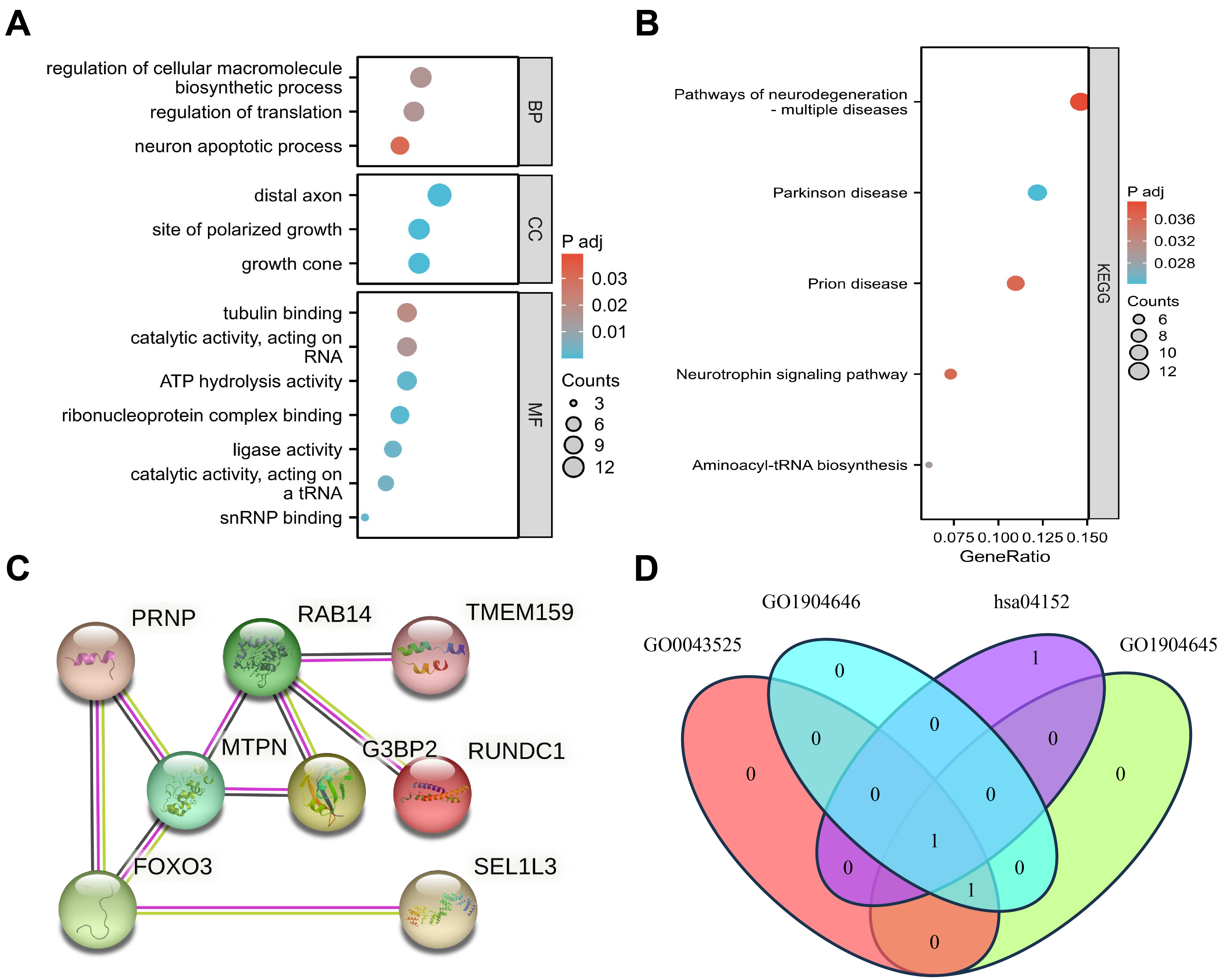

Supplement: Supplementary file 2 — Figure S2. Analysis of ncRNA-seq and selection of genes interacting with miR-301a-3p. Note: (A) Bubble plot of GO analysis of DGEs in RNA-seq data. (B) Bubble plot of KEGG analysis of DGEs in RNA-seq data. (C) PPI network of the 13 DEGs obtained from Venn analysis. (D) Venn analysis shows enrichment of the 13 DEGs in GO and KEGG terms. (n = 3 in each group). (JPG 2126 KB) [file 10565_2024_9865_MOESM2_ESM.jpg]

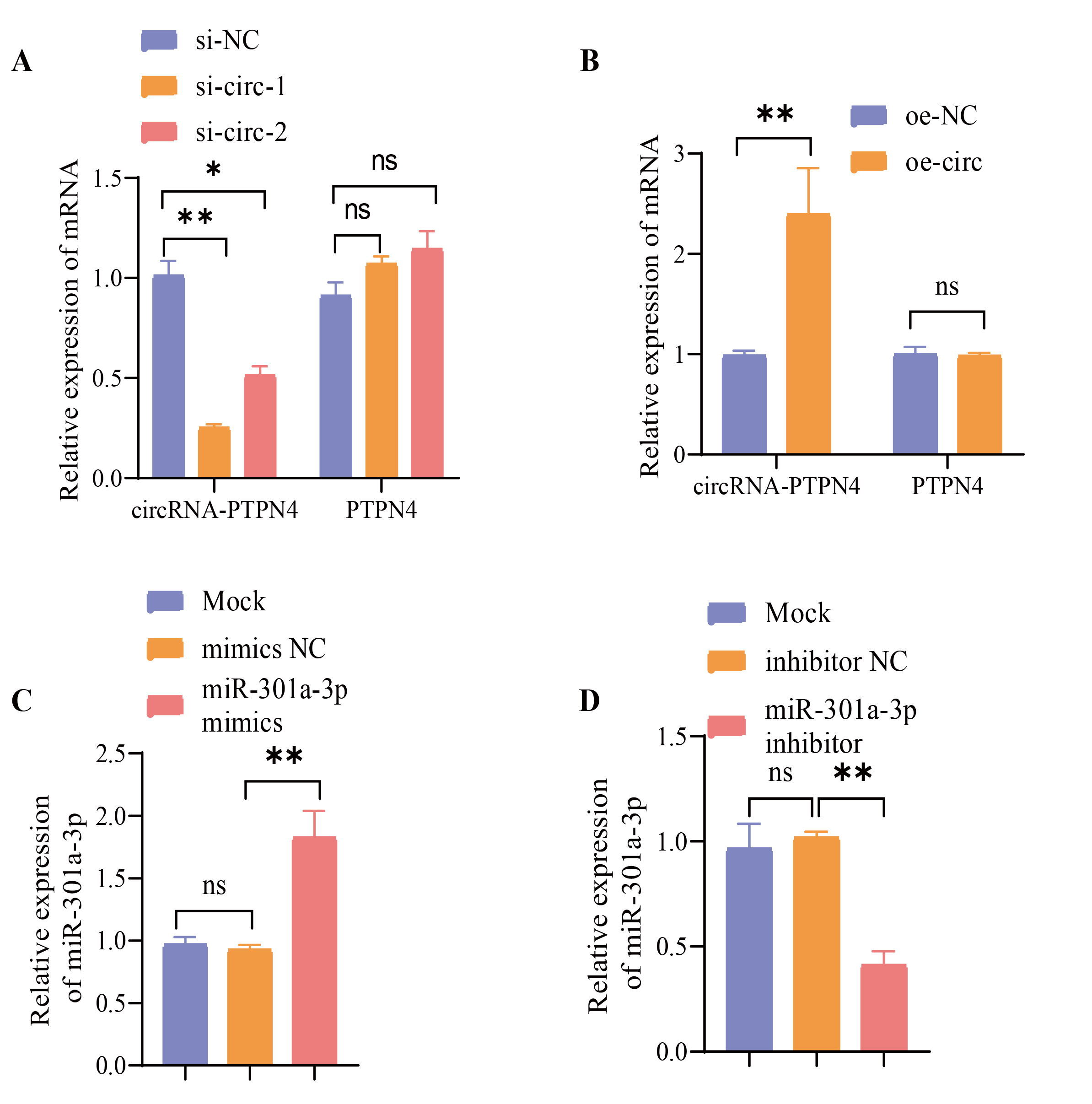

Supplement: Supplementary file 3 — Figure S3. Interaction between miR-301a-3p and FOXO3. Note: (A) Expression changes of circRNA-PTPN4 after circRNA-PTPN4 silencing. (B) Expression changes of circRNA-PTPN4 after circRNA-PTPN4 overexpression. (C) Expression changes of miR-301a-3p after miR-301a-3p mimics treatment. (D) Expression changes of miR-301a-3p after miR-301a-3p inhibitor treatment. ns: not significant (P > 0.05); *: (P < 0.05); **: (P < 0.01); All cell experiments were repeated 3 times. (JPG 568 KB) [file 10565_2024_9865_MOESM3_ESM.jpg]

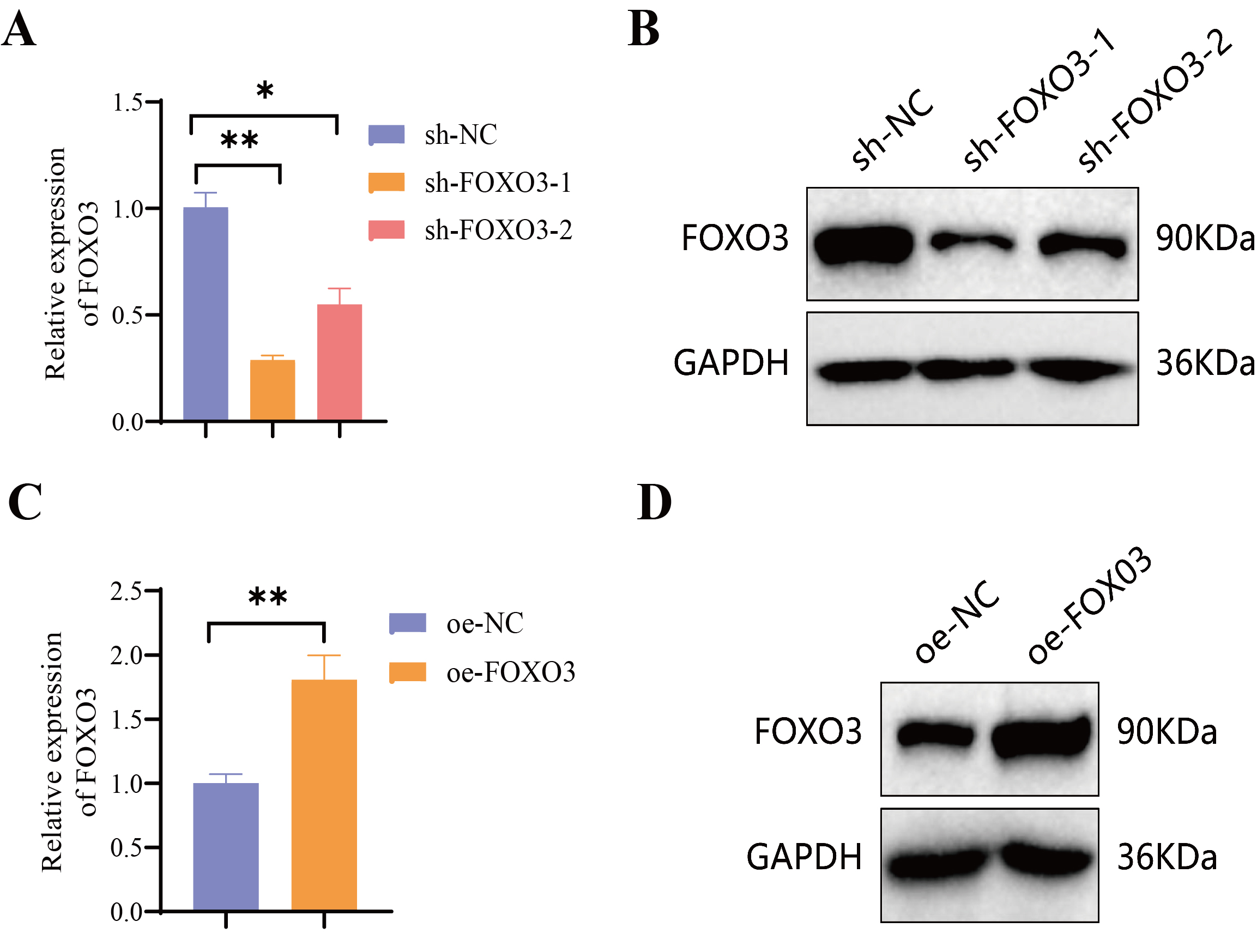

Supplement: Supplementary file 4 — Figure S4. Validation of the effects of FOXO3 silencing and overexpression. Note: (A) Expression changes of mRNA after FOXO3 silencing. (B) Expression changes of protein after FOXO3 silencing. (C) Expression changes of mRNA after FOXO3 overexpression. (D) Expression changes of protein after FOXO3 overexpression. *: (P < 0.05); **: (P < 0.01); All cell experiments were repeated 3 times. (JPG 515 KB) [file 10565_2024_9865_MOESM4_ESM.jpg]
