# Supplementary material for: circRNA-PTPN4 mediated regulation of FOXO3 and ZO-1 expression: implications for blood–brain barrier integrity and cognitive function in uremic encephalopathy
Source: Cell Biol Toxicol. 2024 Apr 17;40(1):22. doi: 10.1007/s10565-024-09865-6 (PMC11024022; doi:10.1007/s10565-024-09865-6)
Supplement: Supplementary file 5 — (DOCX 18 KB) [file 10565_2024_9865_MOESM5_ESM.docx]

**Table S1. Sequence of shRNA and siRNA**

| Name | Sequence (5’-3’) |
| --- | --- |
| sh-NC | CCTAAGGTTAAGTCGCCCTCG |
| sh-FOXO3-1 | GGAGTACATTTGCTGGATTCT |
| sh-FOXO3-2 | ACGGACAGGAGTACATTTGCT |
| si-NC | UUCUCCGAACGUGUCACGUTT |
| si-circRNA-PTPN4-1 | AGCTCTGACTTTGTGTGGACA |
| si-circRNA-PTPN4-2 | AGGAGTCAGCTCTGACTTTGT |

Abbreviations: sh, shRNA, short hairpin RNA; si, siRNA, small interfering RNA.

**Table S2. Sequence of RT-qPCR**

| Gene | Sequence (5’-3’) |
| --- | --- |
| circRNA-PTPN4 | F: CAGCTCTGACTTTGTGTGGA |
|  | R: GGCCAACTCTGATGCTCGTA |
| miR-301a-3p | F: CTCTCGCACATCAUATGCACTAG |
|  | R: universal primer |
| FOXO3 (human) | F: CGGACAAACGGCTCACTCT |
|  | R: GGACCCGCATGAATCGACTAT |
| FOXO3 (mouse) | F: GGGGAACCTGTCCTATGCC |
|  | R: TCATTCTGAACGCGCATGAAG |
| ZO-1 (human) | F: CAACATACAGTGACGCTTCACA |
|  | R: CACTATTGACGTTTCCCCACTC |
| Occludin (human) | F: ACAAGCGGTTTTATCCAGAGTC |
|  | R: GTCATCCACAGGCGAAGTTAAT |
| Claudin-5 (human) | F: CTCTGCTGGTTCGCCAACAT |
|  | R: CAGCTCGTACTTCTGCGACA |
| GAPDH (human) | F: GGAGCGAGATCCCTCCAAAAT |
|  | R: GGCTGTTGTCATACTTCTCATGG |
| GAPDH (mouse) | F: AGGTCGGTGTGAACGGATTTG |
|  | R: GGGGTCGTTGATGGCAACA |
| U6 | F: CTCGCTTCGGCAGCACA |
|  | R: universal primer |

Abbreviations: F, forward; R, reverse.

**Table S3. Primary antibody information**

| Antibody | Cat.NO | Dilution ratio | Manufacturer | Country |
| --- | --- | --- | --- | --- |
| ZO-1 (anti-rabbit) | ab307799 | 1: 1000 | Abcam | UK |
| Occludin (anti-rabbit) | ab216327 | 1: 1000 | Abcam | UK |
| Claudin 5 (anti-rabbit) | ab131259 | 1: 1000 | Abcam | UK |
| FOXO3 (anti-rabbit) | ab314007 | 1: 10000 | Abcam | UK |
| GAPDH (anti-rabbit) | ab181602 | 1: 10000 | Abcam | UK |

**Table S4. Sequence of ChIP-qPCR**

| Name | Sequence (5’-3’) |
| --- | --- |
| P1 | F: AATTACCGTGCAAGCGAGGA |
|  | R: AACAAAAACGAGACGCTGCC |
| P2 | F：CAAACCAACAGGCAGCGTC |
|  | R：TCTCAACCGTTAGCACCCAA |

Abbreviations: F, forward; R, reverse

**Table S5. Information about the GEO datasets**

| Accession | Platform | Summary |
| --- | --- | --- |
| GSE131708 | GPL23365 | Peripheral blood from 4 cases of viral meningitis and 4 healthy controls |
| GSE199759 | GPL18402 | Brain tissue from 16 patients with GRE and 9 patients without GRE |
| GSE86291 | GPL18402 | Plasma samples from 7 patients with acute cerebral infarction and 4 healthy controls |
| GSE195732 | GPL20301 | Plasma samples from 12 DVA patients with CCMs and 6 DVA patients |
| GSE179819 | GPL24158 | Serum samples from 15 patients with sporadic ALS and 16 healthy controls |

Abbreviation: ALS, amyotrophic lateral sclerosis; DVA, developmental venous anomalies; CCMs, Cerebral cavernous malformations; GRE, glioma-related epilepsy

**Table S6. CircRNAs parent genes at circBase**

| CircRNA ID | Annotation | Best transcript | Gene sympol |
| --- | --- | --- | --- |
| mmu_circ_0000069 | ANNOTATED, CDS, coding, INTERNAL | NM_019933 | Ptpn4 |
| mmu_circ_0000047 | ANNOTATED, CDS, coding, INTERNAL | NM_175510 | Unc80 |
| mmu_circ_0000334 | ANNOTATED, CDS, coding, INTERNAL | NM_029606 | Ccdc46 |
| mmu_circ_0000320 | ANNOTATED, CDS, coding, INTERNAL | NM_001080925 | Rapgefl1 |
| mmu_circ_0000547 | ANNOTATED, CDS, coding, INTERNAL, OVCODE, OVEXON | NM_001033272 | Spata13 |

**Table S7. GO/KEGG enrichment analysis**

| Ontology | ID | Description | Pvalue | GeneID | Count |
| --- | --- | --- | --- | --- | --- |
| BP | GO:1904646 | cellular response to amyloid-beta | 0.0003 | PRNP/FOXO3 | 2 |
| BP | GO:0043525 | positive regulation of neuron apoptotic process | 0.0004 | PRNP/FOXO3 | 2 |
| BP | GO:1904645 | response to amyloid-beta | 0.0004 | PRNP/FOXO3 | 2 |
| KEGG | hsa04152 | AMPK signaling pathway | 0.0031 | RAB14/FOXO3 | 2 |
