# Supplementary material for: circRNA-PTPN4 mediated regulation of FOXO3 and ZO-1 expression: implications for blood–brain barrier integrity and cognitive function in uremic encephalopathy
Source: Cell Biol Toxicol. 2024 Apr 17;40(1):22. doi: 10.1007/s10565-024-09865-6 (PMC11024022; doi:10.1007/s10565-024-09865-6)
Supplement: Supplementary file 6 — (PDF 4291 KB) [file 10565_2024_9865_MOESM6_ESM.pdf]

20240318101950967046803592474624

WORD COUNT	7153	TIME SUBMITTED	18-MAR-2024 10:20AM
		PAPER ID	107631907

Abstract

Uremic encephalopathy (UE) poses a significant challenge in neurology, leading to the need for investigating the involvement of non-coding RNA (ncRNA) in its development. This study employed ncRNA-seq and RNA-seq approaches to identify fundamental ncRNAs, specifically circRNA and miRNA, in the pathogenesis of UE using a mouse model. *In vitro* and *in vivo* experiments were conducted to explore the circRNA-PTPN4/miR-301a-3p/FOXO3 axis and its effects on blood-brain barrier (BBB) function and cognitive abilities. The research revealed that circRNA-PTPN4 binds to and inhibits miR-301a-3p, leading to an increase in FOXO3 expression. This upregulation results in alterations in the transcriptional regulation of ZO-1, affecting the permeability of human brain microvascular endothelial cells (HBMECs). The axis also influences the growth, proliferation, and migration of HBMECs. Mice with UE exhibited cognitive deficits, which were reversed by overexpression of circRNA-PTPN4, whereas silencing FOXO3 exacerbated these deficits. Furthermore, the uremic mice showed neuronal loss, inflammation, and dysfunction in the BBB, with the expression of circRNA-PTPN4 demonstrating therapeutic effects. In conclusion, circRNA-PTPN4 plays a role in promoting FOXO3 expression by sequestering miR-301a-3p, ultimately leading to the upregulation of ZO-1 expression and restoration of BBB function in mice with UE. This process contributes to the restoration of cognitive abilities.

Introduction

Uremic encephalopathy (UE) is a severe complication that could arise when chronic kidney disease advances to the uremic stage, predominantly impacting the central nervous system of patients (PMID: 34736971; PMID: 34736971; PMID: 31177383). Common symptoms of this condition include compromised consciousness, malfunctioning motor nerves, and behavioral and cognitive impairments (PMID: 28211668; PMID: 34068165). Although UE substantially impacts the quality of life and prognosis of patients, its exact pathogenesis remains unclear. This lack of clarity presents a challenge in the search for effective treatment methods.

In recent years, there has been progress in the study of non-coding RNA (ncRNA) in biomedicine (PMID: 33973623; PMID: 33860799; PMID: 31493559; PMID: 29170536). Of note, ncRNAs, particularly microRNAs (miRNAs) and circular RNAs (circRNAs), are recognized to confer crucial functions in proliferative, migratory, and apoptotic capabilities of cancers (PMID: 33860799; PMID: 32437725; PMID: 34588660). The roles and mechanisms of ncRNAs in neurological diseases, including UE, have received attention from researchers (PMID: 31743302).

The blood-brain barrier (BBB) is vital in shielding the brain from harmful substances and serves as the principal impediment for drugs to penetrate the brain (PMID: 35568085; PMID: 35387495; PMID: 30280653). Various neurological disorders have been found to cause impairment in the function of the BBB, with UE being particularly notable (PMID: 35387495; PMID: 37169097; PMID: 21611756). Hence, grasping the molecular dynamics influencing the BBB function is essential. Such knowledge is pivotal for delving into the pathogenesis of UE and devising appropriate treatment methodologies.

Based on the background and understanding provided, this study aims to elucidate ncRNA's pivotal role and underlying mechanisms in UE using high-throughput sequencing technology. Specifically, our investigation is centered on exploring the involvement of the circRNA-PTPN4/miR-301a-3p/FOXO3 axis in this process, as well as its contribution to the onset and progression of UE

by affecting the function of the BBB. Through these studies, we aim to provide novel insights and strategies for treating UE, resulting in tangible clinical benefits for patients.

Materials and Methods

Sample Preparation for Sequencing

The isolation of total RNA from brain and kidney tissues of both normal mice (n = 3) and uremic mice (n = 3) was accomplished using Trizol reagent (Thermo). Quantification of RNA, along with assessments of its purity and structural integrity, was conducted through the utilization of a Qubit® 2.0 Fluorometer® in conjunction with a Qubit® RNA Assay Kit for concentration measurements, an IMPLEN Nanodrop spectrophotometer for evaluating RNA purity, and an RNA Nano 6000 Assay Kit employed on an Agilent Bioanalyzer 2100 system for determining the structural integrity of the RNA samples. The measurements yielded results indicating that RNA concentration was equal to or greater than 20 ng/μL, purity exceeded an OD260/280 ratio of 2.0, and integrity showed a RIN value equal to or greater than 7.0 with a 28S/18S ratio equal to or greater than 1.0 (PMID: 34983375).

High-throughput Transcriptome Sample Sequencing

For the preparation of RNA samples, 3 μg of total RNA from each sample served as the input for cDNA library preparation using the NEBNext® Ultra™ RNA Library Prep Kit for Illumina® (NEB, E7435L, Beijing, China) (PMID: 32235747; PMID: 30805932). The construction of a reference genome index was achieved via Bowtie2 version 2.2.8, allowing for the alignment of paired-end purified reads against the reference genome. The identification of established miRNAs was facilitated through the use of corresponding miRNA tags. Additionally, the potential miRNAs were discerned by referencing miRBase 20.0, utilizing the capabilities of mirdeep2 and srna-tools-cli software for this purpose (PMID: 32035423). For circRNA sequencing, after DNA end adenylation and adaptor ligation, cDNA fragments (150-200 bp) were purified with the AMPure XP system and PCR-

amplified, with the final products assessed on the Agilent Bioanalyzer 2100. The indexed samples were then clustered and sequenced on the Illumina HiSeq 4000, generating 150 bp paired-end reads (PMID: 31786335).

Sequencing Data Analysis

The R software package "Limma" was employed for the analysis of miRNAs and circRNAs that exhibited differential expression between control and uremic groups, adopting thresholds of absolute log fold change (|logFc|) greater than 1 and a P-value less than 0.05. For the selection of differentially expressed genes (DEGs), criteria set were an absolute log fold change (|logFC|) exceeding 1 and a P-value below 0.001. The construction of Venn diagrams to display gene overlap utilized the "vennDiagram" package. Functional enrichment analyses, including GO and KEGG pathways, were conducted using the "clusterProfiler" package. Protein-protein interaction (PPI) networks were explored using the STRING database online, while circRNA-miRNA interaction predictions were made via the ERCOI web resource. Predictions of miRNA target genes were facilitated through both the miRmap and Tarbase databases (PMID: 35365629).

***In vitro* Cell Culture**

Human brain microvascular endothelial cells (HBMECs) (CRL-3245) were maintained in a culture medium consisting of DMEM/F12, enriched with 40 µg/ml endothelial cell growth supplement (ECGS) and 10% FBS. Meanwhile, HEK-293T cells (CRL-3216), utilized in this research, were sourced from ATCC, United States. These cells were maintained at optimal growth conditions in a Thermo Fisher incubator set at 37°C with an atmosphere of 5% CO_2. The culture media components, FBS and DMEM/F-12, were acquired from Gibco (PMID: 22278418).

Cell Transfection

Lentiviral particles were packaged in HEK-293T cells via transfection with the target plasmid along with the auxiliary plasmids pMD2.G (12259, Addgene) and psPAX2 (12260, Addgene) using the Lentiviral Packaging Kit (V48820, Invitrogen). The construction of target plasmids was undertaken by Shanghai Hanheng Biotechnology Co., Ltd., with sh-RNA and si-RNA sequences detailed in Table S1.

Transfection of mimics-NC/miR-301a-3p-mimics and inhibitors-NC/miR-301a-3p-inhibitors was performed using lipofection, with both mimics and inhibitors procured from Sangon Biotech (Shanghai, China). The sequences were as follows: mimics-NC: UUGUACUACACAAAAGUACUG; miR-301a-3p-mimics: CAGUGCAAUAGUAUUGUCAAAGC; miR-301a-3p-inhibitors: GCUUUGACAAUACUAUUGCAC.

Cell groups for the experiment were organized as follows: for circRNA-PTPN4: si-NC, si-circ-1, si-circ-2, oe-NC, oe-circ; for miR-301a-3p: Mock, inhibitor NC, miR-301a-3p inhibitor, mimics NC, miR-301a-3p mimics; for FOXO3: sh-NC, sh-FOXO3-1, sh-FOXO3-2, oe-NC, oe-FOXO3, oe-circ+sh-FOXO3, si-circ+oe-FOXO3.

Gene and Protein Expression Profiling

Total RNA was isolated from both tissues and cellular samples utilizing the Trizol reagent provided by Thermo Fisher Scientific, and subsequently reverse-transcribed into cDNA with the aid of the First Strand cDNA Synthesis Kit (D7168L, Beyotime, Shanghai, China). In the case of miRNAs, cDNA synthesis was facilitated through the use of a PolyA Tailing Kit (Sangon Biotech, Shanghai, China), enabling the generation of miRNAs appended with PolyA tails. RT-qPCR was performed using an RT-qPCR Kit (Q511-02, Vazyme Biotech, Nanjing, China) following the manufacturer's instructions. Primer sequences were designed and supplied by Sangon Biotech (Shanghai, China), with details in (Table S2). GAPDH served as the internal reference for mRNA, and U6 for miRNA. Gene expression quantification was achieved using the 2-ΔΔCt method (PMID: 27295416).

For protein extraction from tissues and cells, RIPA lysis buffer containing 1% PMSF (Beyotime) was used. SDS-PAGE gels of 8%-12% were prepared according to the size of the target protein bands, and proteins were separated by electrophoresis. Primary antibodies (Table S3) were added and incubated overnight at 4°C. HRP-conjugated goat anti-rabbit IgG secondary antibody (ab6721, 1:2000, Abcam, UK, and Cell Signaling Technology) was applied and incubated for 1 h at room temperature. The bands were visualized using ECL solution (1705062, Bio-Rad) on an Image Quant LAS 4000C gel documentation system (GE). As normalized to GAPDH, band intensities were quantified using ImageJ software to determine protein levels (PMID: 31002347).

Assessment of Cell Viability, Proliferation, Migration, and Apoptosis

The evaluation of cell viability was conducted utilizing the Cell Counting Kit-8 (CCK-8, Beyotime). Cells, resuspended and adjusted to a density of 1×10^3 per well, were seeded into 96-well plates and cultured overnight. Subsequent to culture intervals of 24, 48, and 72 h, each well received 10 µL of the CCK-8 solution, which was then incubated for an hour prior to the assessment of optical density at 450 nm using a microplate spectrophotometer (E8051, Promega) (PMID: 27506793).

For proliferation rates, the EdU labeling assay was utilized. Following seeding in 24-well plates, cells were treated with 10 µmol/L EdU (Beyotime) and allowed to incubate for 2 h. Subsequently, the cells were immobilized using 4% paraformaldehyde, rendered permeable with 0.5% Triton X-100, and subjected to staining via the EdU click reaction protocol. DAPI staining was applied to visualize nuclei. Fluorescence microscopy (FV-1000/ES, Olympus, Japan) quantified the percentage of EdU-positive cells in random fields (PMID: 35578338).

Wound healing assays were conducted by seeding cells in 6-well plates until reaching 90-100% confluence, followed by creating a scratch with a pipette tip. Images were taken at 24 h to measure migration distances using Image J software, calculating relative migration rates (PMID: 35392845).

TUNEL assays detected apoptosis with the Beyotime TUNEL Apoptosis Detection Kit. Fixed and permeabilized cells were incubated with TUNEL reaction mixture and counterstained with DAPI.

Fluorescent microscopy identified TUNEL-positive cells, and the apoptosis rate was calculated by counting positive cells in five random fields per sample (PMID: 21964436).

Luciferase Activity Assay

The cDNA fragments of circRNA-PTPN4 and FOXO3 containing miR-301a-3p binding sites, along with a DNA fragment of ZO-1 harboring a FOXO3 binding site, were cloned into the pmirGLO vector. Mutated versions of these fragments, synthesized through site-directed mutagenesis, were also inserted into the pmirGLO vector. The sequences for each construct were as follows: circRNA-PTPN4 Wild Type (Wt): AAAACUUCAGCACUGUUGCACUU; circRNA-PTPN4 Mutant (Mut): AAAACUUGUGCUGAGAACGUGAU; FOXO3 Wt: GCCGAGAUCAUGCCAGUGCACUC; FOXO3 Mut: GCCGAGAUCAUGCCAGUGCACUC; ZO-1 Wt: TGTAAACA; ZO-1 Mut: ACATTTGT. Using lipofection, HEK293T cells were co-transfected with either circRNA-PTPN4-Wt/Mut or FOXO3-Wt/Mut recombinant vectors and either mimics NC or mimics miR-301a-3p; ZO-1 Wt/Mut recombinant vectors with either oe-NC or oe-FOXO3. After a 48-h incubation, the Dual-Luciferase® Reporter Assay System (E1910, Promega) facilitated the measurement of reporter gene activity, using Renilla luciferase as an internal reference (PMID: 32377874).

RNA/DNA Pull-down

Biotinylated constructs of circRNA-PTPN4 WT/Mut, FOXO3 WT/Mut, and ZO-1 WT/Mut (KeyGEN BioTECH, Wuhan, China) were transfected into HBMEC cells for 48 h. Cells were harvested, washed with PBS, and lysed. Lysates were incubated with RNase-free BSA and yeast tRNA-precoated streptavidin magnetic beads (Merck, LSKMAGT) at 4°C overnight. Following washes with lysis, low-salt, and high-salt buffers, bound RNAs were purified using Trizol and analyzed for miR-301a-3p and FOXO3 enrichment via RT-qPCR (PMID: 29350287).

ChIP-qPCR

Cells fixed with 1% formaldehyde were sonicated, generating appropriately sized DNA fragments, and centrifuged. Supernatants were incubated with anti-rabbit IgG (negative control) or anti-FOXO3 antibody (Abcam, ab70315, UK) overnight at 4°C. DNA-protein complexes were precipitated, decrosslinked at 65°C overnight, and purified. ChIP-qPCR products were analyzed on a 3% agarose gel, with primer sequences in Table S4 (PMID: 27506793).

Fluorescence in Situ Hybridization (FISH)

Probes for circRNA-PTPN4 and miR-301a-3p were obtained from Sangon Biotech (Shanghai, China). After denaturing and fixation, cells were treated with sodium bisulfite and proteinase K, followed by dehydration through an ethanol series. Post-hybridization overnight at 37°C in a humidified chamber, slides were washed and counterstained with DAPI. Fluorescence microscopy observed the hybridization signals (PMID: 29348628).

Transendothelial Electrical Resistance (TEER) Measurement

TEER was assessed prior to FITC-dextran permeability evaluation, following previously described protocols. Briefly, culture medium in both dishes and Transwells was replaced with 0.1M KCl. The EndOhm chamber's cap was inserted at the top of the chamber, connecting the Transwell with a connector cable, and resistance was measured using an EVOM resistance meter (World Precision Instruments, Sarasota, FL). A new Transwell containing 0.1M KCl, devoid of cells, served as a blank control (PMID: 31101061).

FITC-Dextran Endothelial Permeability Assay

For FITC-dextran permeability assessment, HBMECs in logarithmic growth phase were seeded at a density of 1 × 10^5 cells onto the upper chamber of 24-well Transwell plates (3524, Corning). Each chamber was supplemented with 100 μL and 600 μL of medium, respectively, and incubated at 37°C in a 5% CO_2 incubator. Upon reaching confluence, 1 mg/mL FITC-dextran (Sc-263323, SANTA

CRUZ) was introduced and incubated at 37°C for 5 min in a 5% CO_2 incubator. A 200 µL sample of the baseline medium was taken to determine the baseline value. After an additional 24-h incubation with supplemented medium, another 200 µL of the baseline medium was collected to measure FITC fluorescence intensity using a microplate reader (PMID: 33241954).

UE Mouse Model

A total of 126 male C57BL/6 mice, aged 5-6 weeks (sourced from Vital River Laboratory Animal Technology Co. Ltd., Beijing, China), were maintained under pathogen-free conditions at 26-28°C and 50-65% humidity. To establish a mouse model of UE, an adenine-rich diet was utilized. Mice were initially fed a diet containing 20% casein (Casein code C7906-5G, Sigma) for 7 days, followed by a diet supplemented with 0.2% adenine (Adenine code A0230000, Sigma) mixed with casein for another 7 days to induce tubular injury. This was continued for 7 weeks with a diet containing 0.15% adenine. Control mice were fed a standard diet. After 9 weeks, blood samples from the tail vein were tested using a creatinine assay kit (DICT-500, BioAssay Systems) and a urea nitrogen assay kit (BC1535, Solarbio). Elevated levels of creatinine and urea nitrogen in the uremic mice compared to the normal mice indicated successful model establishment.

From week 8 onwards, mice underwent daily Y-maze and Morris water maze tests to assess their cognitive functions. Upon exhibiting cognitive impairments, the mice were deeply anesthetized with isoflurane and perfused with ice-cold PBS. Brain and kidney tissues were collected for sequencing and histological analysis. Stereotaxic microinjections (Dorsal -0.26 cm, Lateral -0.15 cm, Anterior - 0.02 cm) delivered 4 µL of lentivirus at a titer of 2×10^8 units/mL into the mouse ventricles at a rate of 1 µL/min for 7 consecutive days.

Animal grouping for sequencing involved random assignment into a normal group (Normal) and a UE group (Uremia), with 3 mice in each group. For *in vivo* experiments, mice were divided into the normal group (Normal), UE group (Uremia), UE plus circRNA-PTPN4 overexpression and silence control group (oe-circ + sh-NC), and UE plus circRNA-PTPN4 overexpression and FOXO3 silencing

group (oe-circ + sh-FOXO3), with 30 mice in each group. Fifteen mice from each group underwent Evans blue staining (PMID: 34121452; PMID: 36841828; PMID: 37293711).

Y-Maze Test

The apparatus consisted of a maze with a central starting zone and three branching arms, each measuring 45 cm in length, 10 cm in width, and 15 cm in height, with reward food placed at the end of each arm. Mice were initially allowed to adapt to the experimental setup freely. They were then placed in the starting zone, with their initial positions standardized, and their behavior, including exploration, dwell time, and arm entry frequency, was recorded using a camera. After familiarization with the maze layout and food locations, the testing phase evaluated spatial learning and memory by altering the food's placement to observe if mice could locate the correct position. Data analysis included exploration time, the number of entries into different arms, and the ability to find the reward location (PMID: 34116706).

Morris Water Maze Test

The experiment was onducted in a 120 cm diameter, 50 cm high pool filled with clear, body-temperature water. The test included a 15 cm diameter circular escape platform submerged 2 cm below the water's surface and camouflaged with opaque color. Mice were acclimatized to reduce stress before the trial, involving familiarization with the lab and pool. Experiments commenced from varying starting points around the pool, with mouse behavior meticulously recorded. Training sessions enabled mice to locate the submerged platform, initially marked with cues which were later removed to assess if mice could independently find the platform. Metrics such as escape latency, occupancy in the target quadrant, platform crossings, and total swimming distance were statistically evaluated (PMID: 32451415).

Histological and immunological analysis

For hematoxylin and eosin (H&E) staining, coronal sections of brain tissues, 20 μm thick, were stained using H&E, with incubation of 2 min for hematoxylin and 1 min for eosin. Sections underwent dehydration and permeabilization before microscopic observation (BX63, Olympus, Japan) (PMID: 32358041).

For immunofluorescence assay, fixed cells or tissue sections were treated to allow penetration of primary antibodies targeting ZO-1 (ab307799, Abcam, UK), Occludin (ab216327, Abcam, UK), Claudin-5 (ab131259, Abcam, UK), and CD31 (Sc-376764, SANTA CRUZ, US), followed by incubation with Alexa Fluor-conjugated secondary antibodies and DAPI staining. Confocal microscopy facilitated the visualization of these markers (PMID: 30516406).

The immunohistochemistry protocol entailed the use of antibodies specific to NeuN (ab177487, Abcam), TNF-α (ab307164, Abcam), and IL-1β (ab283818, Abcam). Following secondary antibody application and SABC amplification, DAB chromogen revealed the localization of target proteins, counterstained with hematoxylin. Observations were made under an upright microscope (BX63, Olympus, Japan) (PMID: 32358041).

Evans Blue Assay

To assess vascular leakage, mice were intravenously injected with 100 μL of 2% Evans Blue (E2129, Sigma Aldrich, MO). Then, 2 h post-injection, mice were euthanized and perfused with PBS. Brain tissues were then dissected and examined under a stereo microscope for horizontal and coronal sections. Brain homogenates were centrifuged at 14,000 rpm for 30 min, and the supernatant containing Evans Blue was transferred to a 96-well plate, with 200 μL per well, including replicates. Optical density at 620 nm was measured using a pre-warmed microplate reader (PMID: 36429017).

Statistical Analysis

Bioinformatics results were analyzed using R version 4.2.1, while other analyses were conducted using SPSS version 26.0 (IBM). A P-value < 0.05 was considered statistically significant.

Results

Identification of miR-301a-3p as a Key miRNA Associated with BBB Injury in UE

To examine the molecular mechanisms of miRNAs in BBB injury in UE, we analyzed miRNA expression levels in kidney and brain tissues from normal mice (n = 3) and mice with UE (n = 3) using high-throughput sequencing of ncRNAs (ncRNA-seq). We identified 617 differentially expressed miRNAs (DEMs) in kidney tissue, which we call kidney miRNAs. Among them, 132 miRNAs were downregulated and 485 miRNAs were upregulated, based on the criteria of $|logFc| > 1$ and $P < 0.05$ (Figure 1A). A total of 38 DEMs, referred to as brain-miRNAs, were identified in brain tissue using the criteria of $|logFc| > 0.5$ and $P < 0.05$. Among these miRNAs, 26 were downregulated, while 12 were upregulated (Figure 1B). Subsequently, we performed an intersection analysis on the DEMs from both groups, revealing a total of 7 miRNAs with differential expression common to the kidney and brain tissues of mice with UE (Figure 1C). The expression trends of mmu-miR-323-5p, mmu-miR-301a-3p, and mmu-miR-669f-3p were found to be upregulated in mouse kidney and brain tissues, while mmu-miR-669f-3p showed a downregulation.

To identify the key miRNAs involved in BBB injury in UE, we retrieved BBB injury-related datasets (GSE86291, GSE131708, GSE179819, GSE195732, GSE199759) from the GEO database. Detailed information about these datasets is provided in Table S5. We validated the expression of mmu-miR-323-5p, mmu-miR-301a-3p, and mmu-miR-669f-3p through meta-analysis. The results revealed that miR-301a-3p was the only miRNA expressed in all five datasets. Compared to the Normal group, miR-301a-3p showed upregulation in the BBB injury group, indicating its potential role in the molecular mechanisms of BBB injury (Figure 1D).

Moreover, sensitivity analysis and subgroup analysis were conducted using the one-by-one elimination method on the research results included in this study. The findings indicate that the MD value did not change, suggesting the reliability of the meta-analysis results (Figure 1E; Figure S1A).

The results of bias detection revealed that the data from the meta-analysis were evenly distributed within the funnel plot, suggesting the absence of any publication bias (Figure 2A).

Finally, we conducted calculations to analyze the expression level of miR-301a-3p in the sequencing data. The results demonstrated upregulation of miR-301a-3p expression levels in the kidneys and brain tissues of mice with UE (Figure 2B-C).

These results suggest a high expression of miR-301a-3p in mice with UE.

Effect of the circRNA-PTPN4/miR-301a-3p/FOXO3 Axis on BBB Damage during UE

To explore the regulatory role of miR-301a-3p in BBB damage in UE, we conducted ncRNA-seq and transcriptome sequencing (RNA-seq) on brain tissues obtained from mice with UE. The brain tissue obtained 252 differentially expressed circRNAs (DECs, referred to as brain-circRNAs), consisting of 130 downregulated circRNAs and 122 upregulated circRNAs. These circRNAs met the condition of $|logFc| > 1$ and $P < 0.05$ (Figure 3A). After conditioning on $|logFc| > 1$ and $P < 0.01$, we identified 183 DEGs in brain tissue, which were referred to as brain DEGs. This set included 104 downregulated and 79 upregulated genes (Figure 3B).

We performed an enrichment analysis of 183 DEGs using GO and the KEGG to investigate their enrichment in terms of molecular functions (MF), cellular components (CC), biological processes (BP), and signaling pathways. The results indicated that these DEGs were primarily enriched in regulating biopolymer synthesis and translation. They were also implicated in neuronal apoptosis and signaling pathways associated with neurodegeneration, Parkinson's disease, and neurotrophic factors (Figure S2A-B).

This study aims to identify the circRNA sponge that interacts with miR-301a-3p in UE. We initially used the ERCOI website to predict circRNAs that target miR-301a-3p, which we called target circRNAs. Only 5 circRNAs had matching genes on the website, as shown in Figure S6. A Venn analysis was performed on target circRNAs and brain circRNAs, resulting in the identification of the DEC circRNA-PTPN4, which targets miR-301a-3p in the brain tissue of mice with UE (Figure 3C).

This study aims to identify the specific genes (mRNA) targeted by miR-301a-3p in individuals with UE. We obtained the target genes of miR-301a-3p from the miRmap and Tarbase websites, referred to as miRmap-genes and Tarbase-genes, respectively. Next, the brain-DEGs were compared with miRmap-genes and Tarbase-genes to identify 13 genes targeted by miR-301a-3p in UE (Figure 3D-E). Among these genes, eight showed upregulation in expression, while five showed downregulation. Figure S2C displays the PPI network of 13 DEGs.

To comprehend the enrichment of these 13 genes in the pathogenesis of UE, we carried out GO and KEGG analyses. These 13 genes demonstrated enrichment solely in response to amyloid-like proteins, regulation of neuronal apoptosis, and AMPK signaling pathway entries (Figure 3F; Figure S7). Interestingly, the transcription factor, FOXO3, is consistently enriched in all entries (Figure 3G-H; Figure S2D). These findings suggest that the transcription factor FOXO3 may function as a target gene for miR-301a-3p, which is involved in BBB injury.

Finally, we quantified the expression levels of circRNA-PTPN4 and FOXO3 in the brain tissue of mice with UE. The results demonstrated a downregulation in the expression levels of circRNA-PTPN4 and FOXO3 in mice with UE (Figure 4A-B).

The results above indicate that circRNA-PTPN4 could potentially modulate the expression of FOXO3, leading to the amelioration of BBB impairment in UE through miR-301a-3p sequestration.

Establishing the circRNA-PTPN4/miR-301a-3p/FOXO3 Regulatory Axis in HBMECls

To validate the regulatory relationship between circRNA-PTPN4 and miR-301a-3p, stable transfectants of HBMECs with knockdown of circRNA-PTPN4 (referred to as si-circ) were established. Based on RT-qPCR experiments, neither si-circ-1 nor si-circ-2 affected the expression levels of the parental gene PTPN4 in the cells. Moreover, si-circ-1 demonstrated the most effective silencing effect. Consequently, si-circ-1 (si-circ) was used in subsequent experiments (Figure S3A). The expression level of miR-301a-3p was upregulated, whereas the mRNA expression level of FOXO3 was downregulated following the silencing of circRNA-PTPN4 (Figure 5A). Subsequently,

a stable cell line overexpressing circRNA-PTPN4 was established. The experimental validation results demonstrated successful overexpression of circRNA-PTPN4, which did not impact the expression level of the parental gene PTPN4 within the cells (Figure S3B). Following the overexpression of circRNA-PTPN4, the expression level of miR-301a-3p was diminished, while the mRNA expression level of FOXO3 was increased (Figure 5B).

Subsequently, we utilized the ERCOI website to predict the binding sites between circRNA-PTPN4 and miR-301a-3p. Furthermore, we generated the mutated site sequence of circRNA-PTPN4, as illustrated in Figure 5C. Before experimenting, we validated the overexpression effect of miR-301a-3p mimics (Figure S3C). The luciferase activity assay pointed out a reduction in luciferase activity in the miR-301a-3p mimics group compared to the mimics NC group, while circRNA-PTPN4 Mut was not affected (Figure 5D). The RNA pull-down experiment demonstrated an increase in the enrichment of miR-301a-3p in the Bio-circRNA-PTPN4 Wt group compared to the Bio-NC group.

In contrast, no change was observed in the Bio-circRNA-PTPN4 Mut group (Figure 5E). Additionally, the FISH experiments demonstrated the co-localization of circRNA-PTPN4 and miR-301a-33p in the cytoplasm of cells (Figure 5F). The results above indicate that circRNA-PTPN4 and miR-301a-3p co-localize in the cytoplasm of cells and directly interact with each other.

The effectiveness of the miR-301a-3p inhibitors was verified before the experiment, as depicted in Figure S3D. Compared to the NC group of inhibitors, the miR-301a-3p inhibitors group showed upregulation of FOXO3 mRNA, as indicated by Figure 6A. Next, we treated the cells with miR-301a-3p mimics. Notably, miR-301a-3p mimics treatment resulted in downregulating FOXO3 mRNA compared to the NC group in Figure 6B. We subsequently used the miRmap website to predict the binding site between miR-301a-33p and FOXO3 and then created a mutant sequence of FOXO3 (Figure 6C). The experimental results of the dual-luciferase reporter gene displayed a reduction in luciferase activity of the FOXO3 wild-type group after treatment with miR-301a-3p mimics, in comparison to the mimics NC group. However, no alteration was observed in the FOXO3 mutant group (Figure 6D). The RNA pull-down experiment revealed an increase in the binding of miR-301a-

3p to the Bio-FOXO3 Wt group compared to the Bio-NC group. However, no change was observed in the Bio-FOXO3 Mut group (Figure 6E). Therefore, miR-301a-3p can target and inhibit the expression of FOXO3. Our assays suggest that circRNA-PTPN4 enhances the expression of FOXO3 by binding to miR-301a-3p.

Impact of the circRNA-PTPN4/miR-301a-3p/FOXO3 Axis on BBB Integrity and Endothelial Cell Function in HBMECls

Further investigation is needed to examine the effects of the circRNA-PTPN4/miR-301a-3p/FOXO3 axis on the integrity of the BBB. Initially, the cellular TEER was determined using a TEER analyzer that adheres to the small room transparency model. A FITC-Dextran permeability experiment was also conducted to evaluate cell permeability (Figure 7A). Following the silencing of circRNA-PTPN4, we detected a reduction in TEER and an elevation in fluorescence intensity, suggesting increased permeability and enhanced leakage of large molecules into the basolateral chamber. When circRNA-PTPN4 is silenced, and FOXO3 is overexpressed, the recovery of TEER and the decrease in fluorescence intensity of large molecular permeation into the basolateral chamber indicate a decrease in cellular permeability (Figure 7B-C). When circRNA-PTPN4 is overexpressed, the TEER increases, while the permeability of fluorescence-labeled macromolecules decreases. However, the opposite results are observed when this overexpression is combined with FOXO3 silencing (Figure 7D-E). (The silencing and overexpression effects of FOXO3 were verified before the experiment, as depicted in Figure S4A-D). Considering sh-FOXO3-1 exhibited the most effective silencing, it was selected for subsequent experiments and referred to as sh-FOXO3).

Tight junction (TJ) structures consist of various proteins, primarily transmembrane proteins like Occludin and Claudin-5, and peripheral membrane proteins like ZO-1. Specifically, the ZO-1 protein serves as a crucial connector between the extracellular TJs and intracellular actin, playing a pivotal role in preserving the structural integrity of TJs (PMID: 36497035; PMID: 32697990).

We observed that silencing circRNA-PTPN4 resulted in an upregulation of miR-301a-3p

expression, while the mRNA and protein levels of FOXO3 and ZO-1 were downregulated. Moreover, the expression of other TJ proteins, such as Occludin and Claudin-5, was also reduced. Notably, the overexpression of FOXO3 counteracted the downregulation of these proteins, as demonstrated in Figure 7F-G. Immunofluorescence analysis revealed a notable reduction in fluorescence signals of ZO-1, Occludin, and Claudin-5 in circRNA-PTPN4-silenced cells, accompanied by evident signs of disruption and loss. Overexpression of FOXO3 could enhance the fluorescent signal of these proteins (Figure 7H). Overexpression of circRNA-PTPN4 leads to downregulation of miR-301a-3p expression and enhancement of ZO-1 expression levels and intercellular signaling. Conversely, silencing FOXO3 produces opposite results (Figure 7I-K).

Interestingly, a transcriptional regulatory relationship was unveiled between FOXO3 and ZO-1, which was obtained from the hTFtarget website (Figure 8A). Further experiments based on RT-qPCR, Western blot, and immunofluorescence uncovered a reduction in mRNA and protein levels, consequently disrupting intercellular signaling. In contrast, overexpression of FOXO3 yielded the opposite outcome (Figure 8B-D). Hence, we hypothesize that FOXO3 acts as a transcription factor for ZO-1, controlling its expression and influencing the integrity of TJ structures. To confirm the hypothesis, we utilized the JASPAR website to predict two potential binding sites with high scores between FOXO3 and ZO-1 (Figure 8E). Subsequently, experimental validation of these predicted binding sites was performed. The experimental results from the ChIP-PCR demonstrated the enrichment of FOXO3 at the P1 sequence (Figure 8F). The primary sequence connecting FOXO3 and ZO-1 is the P1 sequence. In subsequent experimental designs, mutated versions of the P1 sequence were used, labeled as ZO-1 Wt and ZO-1 Mut in Figure 8G. Subsequently, we employed biotinylated DNA probes labeled with wild-type ZO-1 (ZO-1 Wt) and mutant ZO-1 (ZO-1 Mut) to conduct DNA pull-down experiments to further substantiate the binding of site 1. The results demonstrate a specific interaction between FOXO3 and ZO-1 Wt. However, no specific interaction was observed between FOXO3 and ZO-1 Mut (Figure 8H). The luciferase reporter, gene experiment results indicate that the oe-FOXO3 group exhibited higher fluorescence enzyme activity of ZO-1 Wt compared to the oe-NCs

group. Conversely, the ZO-1 Mut group showed no change in activity (Figure 8I). The above findings suggest that FOXO3 can enhance the transcription of ZO-1 and modulate the assembly of TJs.

We further examined the alterations in distinct cell populations' growth, proliferation, and migration capacities. Notably, the downregulation of circRNA-PTPN4 led to a decrease in cell growth, proliferation, and migration, accompanied by increased apoptosis. In contrast, the overexpression of FOXO3 had the opposite effect (Figure 9A-D). The overexpression of circRNA-PTPN4 facilitated cell growth, proliferation, and migration while decreasing apoptosis. In contrast, the suppression of FOXO3 yields contrasting outcomes (Figure 9E-H).

The results above demonstrate that the circRNA-PTPN4/miR-301a-3p/FOXO3 axis promotes the formation of TJs in HBMECs by regulating the transcription of ZO-1, thus influencing the functionality of the BBB.

Modulation of BBB Integrity and Cognitive Function by the circRNA-PTPN4/miR-301a-3p/FOXO3 Axis in a Mouse Model of UE

We created a UE mouse model to investigate the impact of the circRNA-PTPN4/miR-301a-3p/FOXO3 axis on the BBB. This model involved lentivirus infection in the cerebral ventricles to simultaneously overexpress circRNA-PTPN4 and silence FOXO3 (Figure 10A). To validate the successful establishment of the model, we performed tests on indicators related to uremia. The results demonstrated elevated creatinine and blood urea nitrogen (BUN) levels in mice with UE compared with normal mice. These findings suggest renal impairment in the model mice (Figure 10B-C).

Subsequently, we evaluated the cognitive function of mice using the Y-maze test and the Morris water maze test. The results indicated a reduction in the percentage of transitions in the Y-maze test within the mice with UE group. However, no statistical difference was observed in total arm entries (Figure 10D). In the Morris water maze experiment, the UE group of mice showed increased escape latency compared to the control group. Additionally, the occupancy rate and the number of target crossings were diminished. However, there was no difference in the total distance traveled. These

findings suggest a decline in cognitive function in mice (Figure 10E). The cognitive function was enhanced in mice from the oe-circ+sh-NC group, whereas cognitive function was diminished in mice following the silencing of FOXO3 (Figure 10D-E).

Following that, we isolated brain tissues from mice. The findings of the RT-qPCR experiment revealed downregulation in the expression levels of circRNA-PTPN4 and FOXO3 mRNA in mice with UE, while the expression level of miR-301a-3p was upregulated. Overexpression of circRNA-PTPN4 resulted in the downregulation of miR-301a-3p expression while upregulating the expression of FOXO3. Conversely, silencing FOXO3 led to the downregulation of circRNA-PTPN4 expression (Figure 10F). These findings suggest that circRNA-PTPN4/miR-301a-3p modulates FOXO3 to impact cognitive function in mice with UE.

Moreover, we performed a pathological analysis and conducted immunohistochemical staining on mouse brain tissue. The H&E staining revealed pronounced cytoplasmic atrophy and neuronal abnormalities in the brains of the mice in the UE model group. The cytoplasm of cells and neurons in the oe-circ+sh-NC group mice returned to normal, while the cytoplasm of cells in the oe-circ+sh-FOXO3 group mice shrank further and neurons displayed abnormalities (Figure 10G). Immunohistochemical staining results of the immune organs revealed a reduction in the NeuN levels, a neuronal marker, in the brains of mice with UE. This reduction indicates the occurrence of neuronal loss in brain tissues. The levels of TNF-α, IL-1β, and other neuroinflammatory biomarkers increased, suggesting the existence of inflammation infiltration in the brain tissue (Figure 11A). In the mice of the group treated with oe-circ+sh-NC, the observed pathological changes and degree of inflammation showed improvement. However, when FOXO3 was silenced, the pathological changes and inflammation worsened (Figure 11A). Subsequently, brain tissue blood vessels were labeled with CD31 using immunofluorescence staining, and the expression of ZO-1 was assessed. The results indicated a reduction in ZO-1 staining in the brains of mice in the UE group compared to the normal group. Following the overexpression of circRNA-PTPN4, the expression of ZO-1 is reinstated, whereas the downregulation of FOXO3 has the converse effect (Figure 11B).

Finally, we assessed the permeability of the BBB in mice by employing Evans Blue staining. The findings demonstrated that mice with UE and the oe-circ+sh-FOXO3 group displayed considerable extravasation of Evans blue, signifying a substantial elevation in the permeability of the BBB. In the circRNA-PTPN4 overexpression group of mice, BBB permeability diminished (Figure 11C).

In conclusion, circRNA-PTPN4 promotes the expression of FOXO3 and upregulates ZO-1 by absorbing miR-301a-3p. This restoration of the BBB function in mice with UE subsequently improves their cognitive abilities.

Discussion

UE is a neurological complication associated with uremia, yet its pathogenesis remains incompletely understood (PMID: 34736971; PMID: 34736971). In recent years, there has been extensive research on 2 the role of ncRNA in the pathogenesis of various diseases. However, its role in UE remains unclear (PMID: 32445090; PMID: 33860799). This study uncovers 58 the crucial role of the circRNA-PTPN4/miR-301a-3p/FOXO3 axis in UE using high-throughput sequencing of ncRNA. This groundbreaking discovery complements existing research and opens new avenues for further disease investigation.

Dysfunction of the BBB has been implicated in the pathogenesis of a range of neurological disorders, underscoring its critical role as a protective interface between the brain and external milieu (PMID: 33208141). Prior research has posited a potential link between UE and compromised BBB integrity, yet the underlying biological mechanisms have yet to be fully elucidated (PMID: 36036353). In this investigation, high-throughput sequencing techniques were employed to reveal an elevated 7 expression of miR-301a-3p in mice with UE (Figure 1-2). Subsequent application of ncRNA-seq and RNA-seq methodologies, alongside predictive analyses from various databases, identified circRNA-PTPN4 as a DEC targeting 68 miR-301a-3p within the cerebral tissues of these mice (Figure 3). The

regulatory interaction among circRNA-PTPN4, miR-301a-3p, and FOXO3 was validated through a series of experiments, including circRNA-PTPN4 silencing and overexpression, dual-luciferase reporter assays, RNA pull-down assays, fluorescent in situ hybridization, and treatments with miR-301a-3p inhibitors (Figure 5-6), indicating a direct interaction and co-localization between circRNA-PTPN4 and miR-301a-3p, thereby facilitating the expression of FOXO3. Further analyses elucidated the impact of the circRNA-PTPN4/miR-301a-3p/FOXO3 axis on the expression and structural integrity of tight junction proteins (Figure 7-9). ZO-1, a pivotal protein in the maintenance of tight junction integrity and thus BBB functionality, acts as a critical bridging molecule between cells, essential for the coherence of cellular junctions in HBMECs (PMID: 36497035; PMID: 32697990). The establishment of a mouse model of UE through intracerebroventricular administration of lentivirus for simultaneous overexpression of circRNA-PTPN4 and silencing of FOXO3 demonstrated that the *in vivo* circRNA-PTPN4/miR-301a-3p/FOXO3 regulatory axis could ameliorate BBB dysfunction and restore cognitive abilities in mice (Figure 10-11). This investigation substantiates the notion that the circRNA-PTPN4/miR-301a-3p/FOXO3 axis plays a crucial role in modulating BBB function by regulating the expression of ZO-1, thus offering new insights into the mechanistic involvement of ncRNAs, particularly circRNAs, in cellular signal transduction and providing a novel model for understanding the complex dynamics of BBB integrity and neurological diseases.

An increasing body of research is focusing on the role of ncRNAs in the etiology and mechanisms of action across various diseases. Studies have identified miR-370-3p in the brain and plasma as a potential biomarker for sepsis-associated encephalopathy (SAE), though not for UE (PMID: 31743302). Similarly, the levels of miR-146a in the brain have been shown to assist in the diagnosis, prognosis, or treatment of transmissible spongiform encephalopathies (TSEs) (PMID: 35359143), while miR-30b can modulate the disease course and immune response in hypoxic-ischemic encephalopathy (HIE) through the regulation of PAI-1 (PMID: 33360521). These findings underscore the significant advantages of circRNAs and miRNAs as molecular markers in the

diagnosis of neurological diseases. Moreover, there is a growing interest in the therapeutic potential of the circRNA-miRNA-mRNA regulatory network. In the field of oncology, He and colleagues observed an upregulation of hsa_circ_0005239 in hepatocellular carcinoma tissues and cell lines. Overexpression of hsa_circ_0005239 could counteract the suppression of the target gene PD-L1 by miR-34a-5p through a ceRNA mechanism, facilitating cellular migration in hepatocellular carcinoma (PMID: 37208963). In the context of neurological injury, research by Yu et al. found that circ-003423 is downregulated in an ox-LDL-induced HBMEC-IM cell model of brain vascular endothelial cell damage. Overexpressing circ-003423 alleviated ox-LDL-induced damage by competitively inhibiting miR-589-5p, thereby mitigating the suppression of TET2 expression and enhancing cellular proliferation and migration (PMID: 34226983). Additionally, Zhou and colleagues demonstrated that circ-EPS15 is downregulated in both Parkinson’s disease (PD) patients and mouse models. Overexpression of circ-EPS15 could absorb miR-24-3p, enhancing PINK1-PRKN-mediated mitophagy, thereby ameliorating neurological damage (PMID: 37014258). In an oxygen-glucose deprivation (OGD)-induced HBMECs model, circ-129657 competitively bound with miR-194-5p to regulate GMFB expression. Silencing circ-129657 upregulated GMFB, promoting endothelial cell proliferation, reducing brain infarct volume, and mitigating neurological damage in MCAO mouse models (PMID: 37073559). While circRNA-miRNA axes have been extensively explored in cancer and neurological diseases such as Alzheimer’s and Parkinson’s, research on their role in UE is sparse.

FOXO3, a transcription factor recognized for its roles in cellular proliferation, programmed cell death, and oxidative stress management (PMID: 33857309; PMID: 35421606; PMID: 35004893; PMID: 33833780), is identified as a critical regulator of the BBB, with particular emphasis on its implications in UE. This research underscores FOXO3's integral function and initiates avenues for examining its neuroprotective effects. While focusing predominantly on molecular dynamics, the outcomes hint at promising therapeutic prospects for managing UE. Notably, adjustments in the circRNA-PTPN4 and FOXO3 expressions emerge as potential therapeutic strategies for this disorder. The investigation provides a comprehensive analysis of ncRNA's involvement in UE, linking it with

BBB functionality (Graphical Abstract). Utilizing high-throughput sequencing alongside bioinformatic techniques, we delineated a key ncRNA regulatory pathway, the circRNA-PTPN4/miR-301a-3p/FOXO3 axis. Laboratory assays underscored the pathway's relevance in modulating cellular permeability, proliferation, and migration. Particularly, circRNA-PTPN4 serves to inhibit miR-301a-3p, facilitating an increased expression of FOXO3, a mechanism corroborated through *in vivo* studies. The UE mouse model illustrated that circRNA-PTPN4 overexpression could significantly enhance cognitive functions, mitigate neuronal loss and inflammation, and ameliorate BBB disturbances. Thus, circRNA-PTPN4 is posited as a protective agent in UE development, laying the groundwork for future investigations into its molecular underpinnings and therapeutic potential.

This research reveals the significant impact of the circRNA-PTPN4/miR-301a-3p/FOXO3 signaling axis on UE progression, providing novel insights into the complex molecular mechanisms characterizing this neurological ailment. By validating the essential influence of ncRNAs, specifically circRNAs and miRNAs, in neurological diseases, the study accentuates the importance of deepening our understanding of ncRNAs in future research. The identification of the circRNA-PTPN4/miR-301a-3p/FOXO3 axis as a viable target opens avenues for innovative therapeutic interventions, promising more precise and efficacious treatment modalities for individuals afflicted with UE. Consequently, the findings introduce new molecular biomarkers for the early detection and prognostic assessment of UE, carrying significant clinical implications that could lead to timely interventions and enhanced patient outcomes.

Over the past decade, research on circRNAs has made significant strides. However, investigations within the domain of neurological diseases have predominantly focused on SAE and HIE, with a relatively unilateral approach towards exploring the mechanisms underlying UE and a scarcity of related studies. This relative void in research hampers the ability to integrate and refine existing studies thoroughly, potentially leading to subjective biases and overlooking pertinent knowledge from adjacent fields. Such limitations could impede the identification and resolution of research gaps and deficiencies, as well as the proposition of novel research directions and

improvements, consequently restricting the depth and breadth of analysis. Moreover, the pathogenesis of UE is recognized as complex, likely resulting from multifactorial contributions, including alterations in hormonal metabolism, retention of uremic solutes, shifts in electrolyte and acid-base balance, blood-brain barrier transport, changes in vascular reactivity, and inflammation, without established diagnostic criteria and with variable clinical presentations (PMID: 33231997). Although mouse models have provided valuable insights, physiological and metabolic differences between mice and humans suggest that findings might not be fully applicable to human cases. Furthermore, while this study centers on the circRNA-PTPN4/miR-301a-3p/FOXO3 axis, the network of ncRNAs is exceedingly complex. Given the broad range of miRNA downstream targets and the complexity of their interactions, other significant ncRNAs may be implicated in the pathogenesis of UE. Although some molecular mechanisms have been uncovered, other unknown pathways and mechanisms related to the progression of UE may exist. Future research needs to validate these findings in larger sample populations and further explore their relevance in humans. Current studies on the association between circRNAs and disease predominantly revolve around the circRNA-miRNA-mRNA regulatory network; however, whether this represents the primary function of circRNAs within organisms remains uncertain.

Future research directions should likely involve comprehensive analyses of circRNAs' interactions with proteins, direct regulation of gene expression, and protein translation, leveraging bioinformatics' big data, and drawing from research methodologies used in miRNA and lncRNA studies to establish an exhaustive and continuously updated circRNA database. This approach will integrate various ncRNA studies for a comprehensive analysis of the circRNA functional network, facilitating deeper investigations into ncRNAs, particularly circRNAs and miRNAs, in UE. This may unveil additional mechanisms and therapeutic targets. Based on this research, the development of therapeutic drugs or strategies targeting the circRNA-PTPN4/miR-301a-3p/FOXO3 axis could commence, offering improved treatment options for patients with UE. In summary, while this study provides new insights into the pathogenesis of UE, further research is necessary to validate these

findings and translate them into clinical applications.

20240318101950967046803592474624

ORIGINALITY REPORT

15%
SIMILARITY INDEX

PRIMARY SOURCES

1	www.frontiersin.org<br>Internet	80 words — 1%
2	link.springer.com<br>Internet	56 words — 1%
3	jneuroinflammation.biomedcentral.com<br>Internet	54 words — 1%
4	molmed.biomedcentral.com<br>Internet	52 words — 1%
5	Sandra Lettlova, Veronika Brynychova, Jan Blecha, David Vrana, Magdalena Vondrusova, Pavel Soucek, Jaroslav Truksa. "MiR-301a-3p Suppresses Estrogen Signaling by Directly Inhibiting ESR1 in ERα Positive Breast Cancer", Cellular Physiology and Biochemistry, 2018<br>Crossref	47 words — 1%
6	www.spandidos-publications.com<br>Internet	39 words — < 1%
7	Xiang Xia, Kundong Zhang, Gang Cen, Tao Jiang, Jun Cao, Kejian Huang, Chen Huang, Qian Zhao, Zhengjun Qiu. "MicroRNA-301a-3p promotes pancreatic cancer progression via negative regulation of SMAD4", Oncotarget, 2015<br>Crossref	38 words — < 1%

8 Shanchun Guo, Pendelton King, Emily Liang, Alyssa A. Guo, Mingli Liu. "LncRNA HOTAIR sponges miR-301a-3p to promote glioblastoma proliferation and invasion through upregulating FOSL1", Cellular Signalling, 2022 — 30 words — < 1%
Crossref

9 Yingmin Wang, Lijuan Gao, Zhili Li, Xingyou Ma. "MicroRNA-301a-3p promotes diabetic retinopathy via regulation of six-transmembrane epithelial antigen of prostate 4", Inflammation Research, 2021 — 30 words — < 1%
Crossref

10 www.science.org — 27 words — < 1%
Internet

11 Ying Wang, Jingchen Du, Yu Liu, Shuhui Yang, Qingshan Wang. "microRNA-301a-3p is a potential biomarker in venous ulcers vein and gets involved in endothelial cell dysfunction", Bioengineered, 2022 — 26 words — < 1%
Crossref

12 www.biorxiv.org — 26 words — < 1%
Internet

13 assets.researchsquare.com — 25 words — < 1%
Internet

14 Jung, W.R.. "Ganglioside GQ1b improves spatial learning and memory of rats as measured by the Y-maze and the Morris water maze tests", Neuroscience Letters, 20080711 — 23 words — < 1%
Crossref

15 Dasgupta, Nupur, You-hai Xu, Ronghua Li, Yanyan Peng, Manoj K. Pandey, Stuart L. Tinch, Benjamin Liou, Venette Inskeep, Wujuan Zhang, Kenneth D.R. Setchell, — 20 words — < 1%

Mehdi Keddache, Gregory A. Grabowski, and Ying Sun. "Neuronopathic Gaucher disease: dysregulated mRNAs and miRNAs in brain pathogenesis and effects of pharmacologic chaperone treatment in a mouse model", Human Molecular Genetics, 2015.
Crossref

16 www.tandfonline.com
Internet
20 words — < 1%

17 Jun Chen, Jian Hu, Huan Liu, Ye Xiong, Yuchi Zou, Wenting Huang, Mingjie Shao, Jiamin Wu, Li Yu, Xiaojie Wang, Xue Wang, Li Lin. "FGF21 Protects the Blood–Brain Barrier by Upregulating PPARγ via FGFR1/β-klotho after Traumatic Brain Injury", Journal of Neurotrauma, 2018
Crossref
19 words — < 1%

18 Shuaihantian Luo, Ruifang Wu, Qianwen Li, Guiying Zhang. "MiR-301a-3p Advances IRAK1-Mediated Differentiation of Th17 Cells to Promote the Progression of Systemic Lupus Erythematosus via Targeting PELI1", Journal of Healthcare Engineering, 2021
Crossref
19 words — < 1%

19 Ting Liu, Ping Ye, Yuanyuan Ye, Sen Lu, Baosan Han. "Circular RNA hsa_circRNA_002178 silencing retards breast cancer progression via microRNA-328-3p-mediated inhibition of COL1A1", Journal of Cellular and Molecular Medicine, 2020
Crossref
18 words — < 1%

20 thejns.org
Internet
18 words — < 1%

21 Fan Zhang, Baoguo Zhang, Rong Tang, Haiping Jiang, Zhimin Ji, Yongjian Chen, Hao Feng. "The
17 words — < 1%

occurrence of lupus nephritis is regulated by USP7-mediated JMJD3 stabilization", Immunology Letters, 2021
Crossref

22 Lei Yang, Huan Liang, Xianguo Meng, Li Shen, Zhanjiang Guan, Bingchang Hei, Haitao Yu, Shanshan Qi. "mmu_circ_0000790 Involves in the Pulmonary Vascular Remodeling in Mice with Hypoxic Pulmonary Hypertension via the microRNA-374c-mediated FOXC1", Molecular Therapy - Nucleic Acids, 2020 — 17 words — < 1%
Crossref

23 bmcgenomics.biomedcentral.com — 17 words — < 1%
Internet

24 pubs.rsc.org — 17 words — < 1%
Internet

25 www.science.gov — 17 words — < 1%
Internet

26 Lingying Liu, Huinan Yin, Xingxia Hao, Huifeng Song et al. "Down-Regulation of miR-301a-3p Reduces Burn-Induced Vascular Endothelial Apoptosis by potentiating hMSC-Secreted IGF-1 and PI3K/Akt/FOXO3a Pathway", iScience, 2020 — 16 words — < 1%
Crossref

27 Liuliu Zhang, Yi Zhang, Huayun Zhu, Xiaofeng Sun, Xiaohua Wang, Pingping Wu, Xinyu Xu. "Overexpression of miR-301a-3p promotes colorectal cancer cell proliferation and metastasis by targeting deleted in liver cancer-1 and runt-related transcription factor 3", Journal of Cellular Biochemistry, 2019 — 16 words — < 1%
Crossref

28 Yixiao Yuan, Xiulin Jiang, Lin Tang, Juan Wang, Qianqian Liu, Xiaolan Zou, Lincan Duan. "SNX20AR/MiRNA-301a-3p/SNX20 Axis Associated With Cell Proliferation and Immune Infiltration in Lung Adenocarcinoma", Frontiers in Molecular Biosciences, 2021 — 16 words — < 1%
Crossref

29 www.jcancer.org — 16 words — < 1%
Internet

30 Mei Zhang, Rong Dong, Jing Yuan, Jingjing Da, Yan Zha, Yanjun Long. "Roxadustat (FG-4592) protects against ischaemia/reperfusion-induced acute kidney injury through inhibiting the mitochondrial damage pathway in mice", Clinical and Experimental Pharmacology and Physiology, 2021 — 15 words — < 1%
Crossref

31 conductscience.com — 14 words — < 1%
Internet

32 translational-medicine.biomedcentral.com — 14 words — < 1%
Internet

33 www.cefe.cnrs.fr — 14 words — < 1%
Internet

34 www.jstage.jst.go.jp — 14 words — < 1%
Internet

35 www.thieme-connect.com — 13 words — < 1%
Internet

36 bmcpulmmed.biomedcentral.com — 12 words — < 1%
Internet

www.sciencedaily.com

37 Internet

12 words — < 1%

38 Changjian Xia, Shifang Li, Wanying Hou, Zaifeng Fan, Hong Xiao, Meiguang Lu, Teruo Sano, Zhixiang Zhang. "Global Transcriptomic Changes Induced by Infection of Cucumber (Cucumis sativus L.) with Mild and Severe Variants of Hop Stunt Viroid", Frontiers in Microbiology, 2017

Crossref

11 words — < 1%

39 Peng-Kai Zhu, Yan-Ping Xu, Ju-Dong Rong, Kai Liu, Tian-You He, Li-Guang Chen, Yu-Shan Zheng, Ling-Yan Chen. "Assessing Genetic Plasticity in Response to New Environmental Conditions in Coniferous Tree Seeds from Multiple Provenances", Research Square Platform LLC, 2024

Crossref Posted Content

11 words — < 1%

40 Yang Cao, Ping Li, Haicun Wang, Lei Li, Quanwang Li. "SIRT3 promotion reduces resistance to cisplatin in lung cancer by modulating the FOXO3/CDT1 axis", Cancer Medicine, 2021

Crossref

11 words — < 1%

41 pericles.pericles-prod.literatumonline.com

Internet

11 words — < 1%

42 Bao, Yonghua, Yongchen Guo, Zexin Li, Wenfeng Fang, Yiqiong Yang, Xuhan Li, Zhuangzhuang Li, Bowen Xiong, Zhiguo Chen, Jianguo Wang, Kang Kang, Deming Gou, and Wancai Yang. "MicroRNA Profiling in Muc2 Knockout Mice of Colitis-Associated Cancer Model Reveals Epigenetic Alterations during Chronic Colitis Malignant Transformation", PLoS ONE, 2014.

Crossref

10 words — < 1%

43 De-Yu Duan, Jing Tang, Hong-Tao Tian, Yang-Yang Shi, Jie Jia. "Adipocyte-secreted microvesicle-derived miR-148a regulates adipogenic and osteogenic differentiation by targeting Wnt5a/Ror2 pathway", Life Sciences, 2021 — 10 words — < 1%
Crossref

44 Lu Li, Rongsheng Zhou, Haigang Lv, Lei Song, Xiaohong Xue, Li Wu. "Inhibitive Effect of Luteolin on Sevoflurane-Induced Neurotoxicity through Activation of the Autophagy Pathway by HMOX1", ACS Chemical Neuroscience, 2021 — 10 words — < 1%
Crossref

45 cslide.ctimeetingtech.com — 10 words — < 1%
Internet

46 www.springermedizin.de — 10 words — < 1%
Internet

47 Anthony, Nicholas. "Identification and Characterization of Novel microRNA Regulators of Myogenesis.", McGill University (Canada), 2021 — 9 words — < 1%
ProQuest

48 Ilona, Gróf. "Tenyészetes Epitélsejt Modellek Alkalmazása Biológiailag Aktív Peptidek és Adjuváns Terápiás Szerek Hatásának Vizsgálatára", Szegedi Tudomanyegyetem (Hungary), 2023 — 9 words — < 1%
ProQuest

49 Qiuchen Zhao, Zhanyang Yu, Fang Zhang, Lena Huang, Changhong Xing, Ning Liu, Yun Xu, Xiaoying Wang. " 3 inhibition prevents oxygen glucose deprivation/reoxygenation-induced transendothelial permeability by elevating γ activity ", Journal of Neurochemistry, 2018 — 9 words — < 1%

Crossref

50 Shao-hua Luan, Yu-qing Yang, Man-ping Ye, Hui Liu, Qiu-fan Rao, Jin-ling Kong, Fan-rong Wu. "ASIC1a promotes hepatic stellate cell activation through the exosomal miR-301a-3p/BTG1 pathway", International Journal of Biological Macromolecules, 2022 — 9 words — < 1%
Crossref

51 Yun Jiang, Jian-yi Wang, Zhi-tian jiang, Jing-Wen Cai et al. "LCN2 attenuates sepsis-induced liver injury by alleviating PTGS2-mediated Ferroptosis", Research Square Platform LLC, 2023 — 9 words — < 1%
Crossref Posted Content

52 patents.google.com — 9 words — < 1%
Internet

53 ricerca.univaq.it — 9 words — < 1%
Internet

54 www.readkong.com — 9 words — < 1%
Internet

55 www.researchsquare.com — 9 words — < 1%
Internet

56 Haibin Liu, Shengtao Sun, Bing Liu. "Smurf2 exerts neuroprotective effects on cerebral ischemic injury", Journal of Biological Chemistry, 2021 — 8 words — < 1%
Crossref

57 Jifeng Yu, Yingmei Li, Ling Sun, Lijie Han, Yu Liu, Danfeng Zhang, Haizhou Xing, Xinsheng Xie, Dingming Wan, Zhongxing Jiang. "SPOP accelerates acute myeloid leukemia initiation and development through miR-183-mediated METAP2 inhibition", Research Square, 2020 — 8 words — < 1%
Crossref

58 Jinhua Hu, Jiugen Ruan, Xiaohong Liu, chijin Xiao, Jianping Xiong. "MicroRNA-301a-3p suppressed the progression of hepatocellular carcinoma via targeting VGLL4", Pathology - Research and Practice, 2018
Crossref
8 words — < 1%

59 Pu, H.. "Limited role of COX-2 in HIV Tat-induced alterations of tight junction protein expression and disruption of the blood-brain barrier", Brain Research, 20071212
Crossref
8 words — < 1%

60 Xiangliang Zhang, Hui Li, Tiantian Zhen, Yu Dong, Xiaojuan Pei, Huijuan Shi. "Hsa_circ_001653 Implicates in the Development of Pancreatic Ductal Adenocarcinoma by Regulating microRNA-377-Mediated HOXC6 Axis", Molecular Therapy - Nucleic Acids, 2020
Crossref
8 words — < 1%

61 Xiao-xu Huang, Qiang Zhang, Hao Hu, Yan Jin, Ai-liang Zeng, Ya-bin Xia, Li Xu. "A novel circular RNA circFN1 enhances cisplatin resistance in gastric cancer via sponging miR-182-5p", Journal of Cellular Biochemistry, 2020
Crossref
8 words — < 1%

62 Xiu-guo Han, Lin Du, Han Qiao, Bing Tu, Yu-gang Wang, An Qin, Ke-rong Dai, Qi-ming Fan, Ting-ting Tang. "CXCR1 knockdown improves the sensitivity of osteosarcoma to cisplatin", Cancer Letters, 2015
Crossref
8 words — < 1%

63 daneshyari.com
Internet
8 words — < 1%

64 journals.plos.org
Internet
8 words — < 1%

65	researcherslinks.com<br>Internet	8 words — < 1%
66	topsecretapiaccess.dovepress.com<br>Internet	8 words — < 1%
67	Hui-Zi Li, Xiang-He Xu, Nan Lin, Da-Wei Wang, Yi-Ming Lin, Zhong-Zhen Su, Hua-Ding Lu. "Overexpression of miR-10a-5p facilitates the progression of osteoarthritis", Aging, 2020<br>Crossref	7 words — < 1%
68	Jingjing Hou, Mei He, Qiang Chen, Siwei Liang. "LncRNA H19 acts as miR-301a-3p sponge to alleviate lung injury in mice with sepsis by regulating Adcy1", Immunopharmacology and Immunotoxicology, 2022<br>Crossref	7 words — < 1%
69	Peerapat Visitchanakun, Pattarin Tangtanatakul, Ornjira Trithiphen, Wipasiri Soonthornchai et al. "Plasma miR-370-3P as a Biomarker of Sepsis-Associated Encephalopathy, the Transcriptomic Profiling Analysis of Microrna-Arrays From Mouse Brains", Shock, 2020<br>Crossref	7 words — < 1%
70	Qiuchen Zhao, Fang Zhang, Zhanyang Yu, Shuzhen Guo, Ning Liu, Yinghua Jiang, Eng H. Lo, Yun Xu, Xiaoying Wang. "HDAC3 inhibition prevents blood-brain barrier permeability through Nrf2 activation in type 2 diabetes male mice", Journal of Neuroinflammation, 2019<br>Crossref	7 words — < 1%
71	Xin Chen, Sai Zhu, Si-Yu Chen, Jia-Nan Wang et al. "miR-301a-3p promotes hepatic stellate cells activation and liver fibrogenesis via regulating PTEN/PDGFR-β", International Immunopharmacology, 2022<br>Crossref	7 words — < 1%

72 Jian-Ping Zhang, Wei-Jing Zhang, Miao Yang, Hua Fang. "Propofol attenuates lung ischemia/reperfusion injury though the involvement of the MALAT1/microRNA-144/GSK3β axis", Molecular Medicine, 2021
Crossref
6 words — < 1%

73 Li Liang, Dachao Zheng, Chao Lu, Qinghong Xi, Hua Bao, Wengfeng Li, Yufei Gu, Yuanshen Mao, Bin Xu, Xin Gu. "Exosomes derived from miR-301a-3p-overexpressing adipose-derived mesenchymal stem cells reverse hypoxia-induced erectile dysfunction in rat models", Stem Cell Research & Therapy, 2021
Crossref
6 words — < 1%

74 Neng Wang, Dan Zhong, Jie Lin, Mei Ye, Yu Chen, Lili Wang, Mei Chen, Cong Luo. "MiR-370-3p aggravates blood–brain barrier injury and neuron apoptosis by targeting SMURF1 to activate the TLR4/MyD88/NF-κB signaling in sepsis-associated encephalopathy", Molecular & Cellular Toxicology, 2022
Crossref
6 words — < 1%

75 Xiang Xia, Shuchang Wang, Bo Ni, Shunpeng Xing, Hui Cao, Zizhen Zhang, Fengrong Yu, Enhao Zhao, Gang Zhao. "Hypoxic gastric cancer-derived exosomes promote progression and metastasis via MiR-301a-3p/PHD3/HIF-1α positive feedback loop", Oncogene, 2020
Crossref
6 words — < 1%

76 Yu-huan Cui, Xiao-qing Zhang, Nai-dong Wang, Mao-dong Zheng, Juan Yan. "Vitexin protects against ischemia/reperfusion-induced brain endothelial permeability", European Journal of Pharmacology, 2019
Crossref
6 words — < 1%

EXCLUDE QUOTES	OFF	EXCLUDE SOURCES	OFF
EXCLUDE BIBLIOGRAPHY	ON	EXCLUDE MATCHES	OFF
